# Experimental and archaeological data for the identification of projectile impact marks on small-sized mammals

**DOI:** 10.1038/s41598-020-66044-3

**Published:** 2020-06-04

**Authors:** Rossella Duches, Nicola Nannini, Alex Fontana, Francesco Boschin, Jacopo Crezzini, Marco Peresani

**Affiliations:** 1 MUSE - Museo delle Scienze (MUSE), Corso del Lavoro e della Scienza 3, IT, 38123 Trento, Italy; 2 Università degli Studi di Siena, Dipartimento di Scienze Fisiche, della Terra e dell’Ambiente, UR Preistoria e Antropologia, Via Laterina 8, IT, 53100 Siena, Italy; 3 Università degli Studi di Ferrara, Dipartimento di Studi Umanistici, Sezione di Scienze Preistoriche e Antropologiche, Corso Ercole I d’ Este 32, IT, 44121 Ferrara, Italy

**Keywords:** Ecology, Environmental social sciences

## Abstract

The role of small game in prehistoric hunter-gatherer economy is a highly debated topic. Despite the general assumption that this practice was uneconomic, several studies have underlined the relevance of the circumstance of capture – in terms of hunting strategies and technology – in the evaluation of the actual role of small mammals in human foraging efficiency. Since very few studies have focused on the recognition of bone hunting lesions, in a previous work we explored the potential of 3D microscopy in distinguishing projectile impact marks from other taphonomic marks, developing a widely-applicable diagnostic framework based on experimental data and focused on Late Epigravettian projectiles. Even though we confirmed the validity of the method on zooarchaeological remains of large-sized mammals, the reliability of the experimental record in relation to smaller animals needed more testing and verification. In this report we thus present the data acquired through a new ballistic experiment on small mammals and compare the results to those previously obtained on medium-sized animals, in order to bolster the diagnostic criteria useful in bone lesion identification with specific reference to small game. We also present the application of this renewed methodology to an archaeological context dated to the Late Glacial and located in the eastern Italian Alps.

## Introduction

The role of small game in prehistoric hunter-gatherer economy is a highly debated topic. Although the exploitation of small prey was not uniform across time and space, their inclusion in the hominin diet is well documented in the Mediterranean area since the Middle Pleistocene^1–10^. A more general trend towards a broad-based economy, consisting of increased diversification of animal resources and greater inclusion of small game in the diet (i.e. Broad Spectrum Revolution; after Flannery 1969^11^), is attested however only at the end of the Pleistocene during the Late Glacial. This process, documented in most of Mediterranean Europe and southern Levant, has been extensively debated and studied at different scales, searching for consistent chronology and causes that could clarify such a synchronous and widespread change in human behaviour^12,13^. Contrary to expectations, as studies in the fields increased, an underestimated complexity of this phenomenon has been revealed. Several factors have been called into question - including demographic pressure, social stress, climatic shifts, changes in subsistence patterns, technological innovations and resource depression - and their relevance has been tested case by case^14–29^.

Investigating shifts in game exploitation from the perspective of evolutionary ecology and, specifically, through the application of the prey choice model^30,31^, the practice of small mammal hunting appears uneconomic regardless of taxa differentiation. Since prey items are ranked according to their energy return balance (i.e. a measure of an animal’s caloric or nutritional value per unit of time taking into account search and post-encounter processing costs), small-bodied and difficult-to-catch mammals are usually categorised as low-ranking resources^30–32^. Going beyond this generalisation, several studies underlined other variables, such as circumstance of capture, hunting technology and game catchability, could be relevant in the evaluation of the actual role of small mammals in human foraging efficiency^33–36^. Moreover, mass collecting appears to be the most economically profitable method of capture for this kind of resource^37–40^. Determining the modality of capture, in terms of hunting strategies and technology, is thus of critical importance for understanding changes in human diets and shifts in game exploitation during Prehistory.

Zooarchaeological and taphonomic studies have made important contributions toward the reconstruction of the dynamic interactions between humans and their prey, primarily defining the criteria useful in the identification of the accumulation agents of a faunal assemblage (through age structure models, anatomical representation, bone breakage patterns and bone surface modifications)^41–57^. Conversely, very few studies have focused on the recognition of projectile impact marks (PIMs; after O’Driscoll and Thompson^58,59^), although they represent the only taphonomic evidence directly connected to hunting technologies employed by humans. Works documenting a few or single archaeological evidence of impact, such as stone fragments embedded in bone or healed wounds, are more numerous^60–79^. Despite some experimental works gave new insights towards the development of a methodology in PIMs recognition^58,80–88^, the recent application of high-resolution quantitative methods in bone taphonomy have mostly addressed cut mark, carnivore tooth marks and trampling marks characterisation^89–106^.

As such, in previous work we used 3D microscopy for the development of a diagnostic method aimed at the distinction of bone hunting injuries from other taphonomic marks: the experimental PIMs taken as reference were produced exclusively by Late Epigravettian projectiles, shaping a widely-applicable framework useful for the identification of hunting bone lesions in other Late Glacial cultural complexes^107^. Even though we confirmed the validity of the method on zooarchaeological remains of large-sized mammals^108^ the reliability of the experimental record in relation to smaller animals needed more testing and verification. Bone dimension and thickness could indeed affect their resistance to projectile impacts, influencing the morphometry of hunting injuries and the representativeness of PIM classes.

In this report we thus present the data acquired through a new ballistic experimentation on small mammals (*Myocastor coypus*) and their comparison to results previously obtained on medium-sized animals, in order to adjust the diagnostic criteria useful in PIMs identification with specific regard to small game. We also present the application of this renewed methodology to an archaeological context dated to the Late Glacial and located in the eastern Italian Alps. This site, named Riparo I of Grotte Verdi di Pradis (hereafter Pradis Cave), represents an optimal case-study, being interpreted as a marmot specialised hunting camp, occupied seasonally by Late Epigravettian hunter-gatherers for the exploitation of this animal resource.

### Methodological background: experimental data and diagnostic criteria in projectile impact mark identification

The previous ballistic experimentation conducted by the authors was aimed at the distinction of hunting lesions caused by Late Epigravettian projectiles on medium-sized ungulates^107^. More specifically, a total of 70 hunting injuries on bones were generated on five complete carcasses by 160 arrows equipped with lithic backed points and backed (bi)truncated bladelets. Each lesion was classified by following the terminology proposed by O’Driscoll and Thompson^58^ and analysed, when possible, through 3D digital microscopy, acquiring multiple morphometrical parameters useful for statistical processing and comparison^107^.

The main methodological result consists of the recognition of drag and puncture marks as the only diagnostic categories in PIMs identification. Critical for their interpretation are the following elements: a) the location of the mark; b) the presence of flaking and/or cracking as secondary feature of both categories; c) the occurrence of specific morphometrical values, different for drags and punctures (detailed below); d) the presence of embedded stone characterised by diagnostic impact fractures^109–117^.

Since drag marks were the most frequent experimental bone injury (50%), they are assumed to be the most likely preserved on zooarchaeological material. This category is mainly located on ribs (40%), vertebrae (34.3%) and long bones, such as the radius and ulna (14.3%). The drag profile is extremely sharp and clean, especially on the most resistant bones, with frequent unilateral flaking (48.6%) and cracking (34.3%)^107^. The distinction of drags from carnivore tooth marks and cut marks (produced both by unmodified flakes and retouched implements) was tested through a 3D microanalysis, considering the parameters listed in the analytic method section (see also Supplementary Fig. S1). Drags turned out to be significantly different from other taphonomic marks, especially regarding some parameters like the breadth at the cut floor (considerably wider than cut marks whilst comparable to that of tooth marks), the depth of cut (considerably higher than other taphonomic marks), the RTD index (Ratio between the breadth at the Top and the Depth of cut; significantly different suggesting a more U-shaped profile than other taphonomic marks) and the opening angle (narrower and more standardised in drags, ranging from 65.8° to 94.5°) (raw data and statistical analysis in Duches *et al*.^107^).

Puncture marks, recorded on the entire skeleton, were the second most frequent experimental trauma (24.3%). When the projectile caused the complete perforation of the bone, an imprint characterised by a more or less regular polygonal outline was generated. Cracking occurred in 48.1% of the punctures. Punctures were compared to carnivore pits and corrosion cavities considering the following parameters acquired through the 3D microanalysis: opening area (A), volume (V), depth of cavity (D) and the ratio between the opening area and volume of pits (RAV)^107^. Puncture marks appeared significantly deeper than carnivore tooth pits and were defined by a statistically lower RAV^107^. The distinction of impact punctures from corrosion cavities was easily conducted considering some morphological features such as the contour of the opening area and the shape of the slopes and floor. This experiment showed that some peculiar features of both drags (sharp edges and polygonal shape) and punctures (polygonal outline) were strictly connected to Late Epigravettian projectiles, confirming the hypothesis of a significant relationship between tip design and PIMs morphometrical features^107^.

To sum up, new clues in PIMs identification and distinction from other taphonomic marks were inferred through the application of 3D digital analysis, qualifying drags and punctures as reliable archaeological indicators of prey capture modalities.

## Results

### Results of experimental activity and comparison to medium-sized mammal experimentation

A total of 90 arrows armed with Late Epigravettian lithic backed points and bladelets were shot against 8 coypu fresh carcasses (Supplementary Fig. S2). The projectiles thoroughly pierced the animal tissues from side to side in 62.3% of cases, whereas arrows which caused a less invasive penetration were far less frequent (n = 27, 35.1%). Only two arrows ricocheted (2.6%), hitting the scapular girdle just beneath the animal skin. The 77 target hits generated a total of 66 hunting injuries on bones. It is noteworthy that, in proportion to the number of hits, more PIMs were produced in this experiment than that obtained on medium-sized ungulates (Supplementary Table S3). Moreover, a higher number of hunting lesions per bone impact were counted (Supplementary Table S3). Both these data are certainly connected to the fragility of coypu bones and their smaller bodies in comparison with medium-sized ungulates.

**Figure 1:**
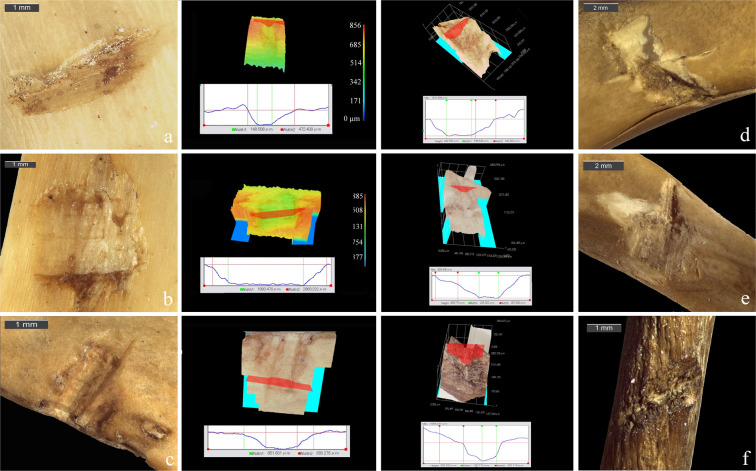
3D cross sections and stereomicroscope images of experimental drag marks on mouflon (**a**,**b**,**c**) and coypu bones (**d**,**e**,**f**) made by Late Epigravettian lithic projectiles.

Five formal categories were identified: drag, drag/fracture, puncture/fracture, puncture with stone embedded and fracture (Table 1). Overall, fractures were the most frequent type of trauma (n = 38, 57.6%), located mainly in the thoracic area, affecting ribs (50%) and vertebrae (15.8%), and less frequently in the head (15.8%) (Table 1 and Supplementary Fig. S4). Drag marks were the second most frequent category (n = 21, 31.8%), distributed homogeneously on the entire skeleton. A different distribution of this kind of trauma could be observed depending on carcass position (Supplementary Fig. S4): the impacts against standing animals produced drag marks on the upper limbs and the thoracic area whereas, on carcasses positioned on four legs, drags resulted in a more uniform distribution with cases on the lumbar vertebrae, the pelvic girdle and the hind limbs. Except for a few specimens characterised by the total removal of a bone edge (1 on femur and 2 on coxal), all drags showed internal microstriations at the base and sometimes on the walls of the groove (Fig. 1 and Supplementary Fig. S5). In 62% of cases, marks were characterised by unilateral or bilateral flaking; two items exhibited cracking and only one showed feathering (for the classification of these secondary traits see O’Driscoll and Thompson^58^). It is also noteworthy that in 19.1% of cases (n = 4) small lithic fragments remained embedded in the groove. Lastly, drags documented on coypu incisors (n = 3; Supplementary Fig. S5) showed extremely sharp and clean profiles. There were only 7 impact punctures (10%), located mostly on pelvic girdle and hind limbs; they were always associated with fractures (n = 4) or stone embedment (n = 3) and were distinguished by a more or less polygonal outline due to the imprint left by the lithic projectile (Supplementary Fig. S6).

**Table 1:** Summary of experimental PIMs location on medium-sized mammals (*Ovis musimon*) and on small-sized mammals (*Myocastor coypus*).

	Drag	Puncture	Fracture	Total	Total %
**Experimentation on medium-sized mammal**					
Head	2	1	1	4	4.3
Vertebrae	12	9		21	22.8
Rib	14	7	1	22	23.9
Scapular girdle	1	8		9	9.8
Front limb	5	4	1	10	10.9
Pelvic girdle	1	1		2	2.2
Hind limb		2		2	2.2
Total	35	32	3	70	100.0
Total %	50.0	45.7	4.3	100.0	
**Experimentation on small-sized mammal**					
Head	3		6	9	9.8
Vertebrae	2	1	6	9	9.8
Rib	5		19	24	26.1
Scapular girdle			3	3	3.3
Front limb	4	1	1	6	6.5
Pelvic girdle	5	3	3	11	12.0
Hind limb	2	2		4	4.3
Total	21	7	38	66	100.0
Total %	31.8	10.6	57.6	100.0

Since few puncture marks were produced in comparison with a very high number of impact fractures and drags, the results confirm the affection of PIM class representativeness by bone dimension and thickness. Moreover, considering that impact fractures are almost indistinguishable from other kind of fractures, this new experimentation established that only drag marks are relevant for the identification of PIMs on zooarchaeological remains of small mammals.

**Figure 2:**
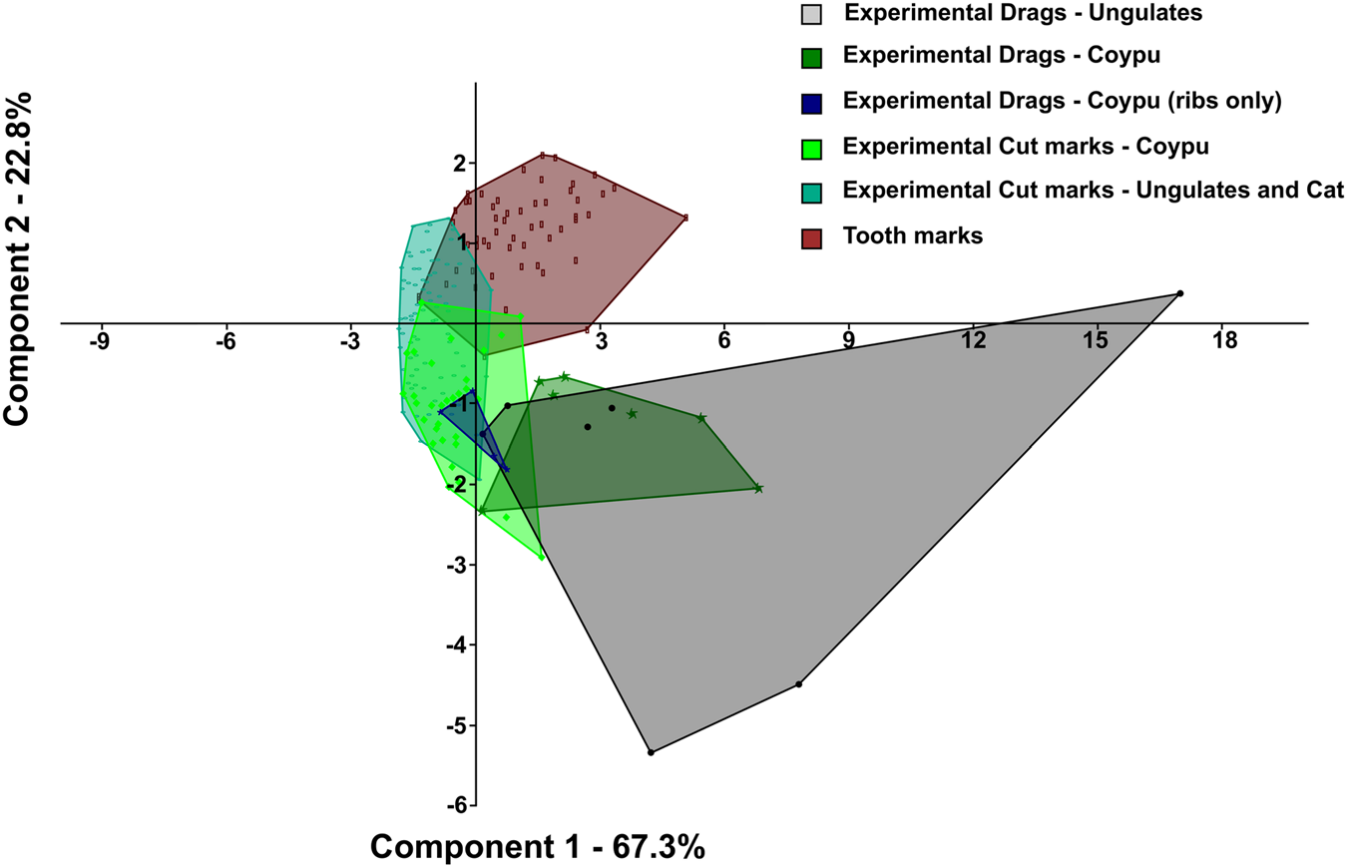
Outcome of a Principal Component Analysis carried out on the considered experimental samples of drags, cut marks and actual carnivore tooth scores (for sample details see the methods section). Parameters used for the test are: DC, BF, BT, SD, GD and OA (Supplementary Table S8 online).

Given that punctures could not be acquired in 3D because of some limitations (presence of stone embedded or discontinuity of the surface), the morphometric analysis was focused only on drags. The profiles and morphometric parameters of experimental drags were significantly different from the features of experimental cut marks produced on coypus as a reference (Supplementary Fig. S7), especially with regards to the depth of cut (Mann-Whitney U-test: U = 10; p = 1.6E-04), breadth at the floor (Mann-Whitney U-test: U = 10; p = 3.0E-04), breadth at the top (Mann-Whitney U-test: U = 10; p = 2.7E-04) and RTF index (Radio between the breadth at the Top and the breadth at the Floor of marks; Mann-Whitney U-test: U = 10; p = 4.3E-02). Coypu drags were generally more U-shaped and characterised by a higher depth of cut, a wider breadth at the floor and a wider breadth at the top (Fig. 1). In order to compare them with drags obtained on medium-sized mammals, a Principal Components Analysis was performed on all the measurements acquired (raw data in Supplementary Table S8). Experimental cut marks and actual carnivore tooth scores were taken as control groups (see methods section and Supplementary Table S8). The general consistency of drag morphometric data coming from the two experiments show that this mark’s features was not influenced by bone size and thickness (Fig. 2). The only exception was drags recorded on ribs, which clearly fell outside PIMs variability, overlapping instead with that of cut marks. This result led us to consider cautiously anthropic marks found on this skeletal element, when analysing archaeological material.

### Application of the methodology to the archaeological case-study

Late Glacial sites of the Alpine area interpreted as specialised marmots hunting camps represent an optimal case-study^21,118–121^. Pradis Cave (SI text and Supplementary Table S9 and Fig. S10) has yielded thousands of marmot bones (*Marmota marmota*), representing about 99% of the whole faunistic assemblage. The number of identified marmot specimens amounts to 11,285 fragments, with the minimum number of individuals being 637^122^ (Table 2 and Supplementary Table S11). The strong prevalence of adult individuals (>13 months, 81%; Supplementary Table S12), the low percentage of carnivore tooth marks (2.8%) and the complete absence of digested remains suggest humans to be responsible for the accumulation (Table 2). Despite differences in the relative abundance of certain skeletal elements, the overall anatomical representation indicates that whole carcasses were introduced to the site. In addition, the strong under-representation of the *autopodium* elements suggests the removal of the fur and its transport elsewhere. Taphonomic analysis confirmed the anthropic exploitation of this animal, thanks to the identification of over 1,200 remains with cut marks (10.8%), located on all skeletal areas (Table 2). Skinning, evisceration, dismembering and intensive defleshing are well represented^122^ (Supplementary Fig. S13). Some percussion marks on long bones (n = 6) suggest the exploitation of the marrow too. Burned remains amount to 6.8% of the samples and concern mainly the long bones.

**Table 2:** Pradis Cave. Marmot anatomical representation with number of PIMs, percentages of cut marks, burned remains and carnivore tooth marks.

	NISP	PIMs	% cut marks	% burned	% carnivore marks
Head	4,468	2	12.3	6.3	0.9
Vertebrae	678		6	2.5	1.7
Rib	878		7,8	1.4	
Scapular girdle	806		22	5.7	3.8
Front limb	1,998	16	11.8	13.7	5.6
Pelvic girdle	651	4	5.8	8.9	8.7
Hind limb	1,533	6	16.4	12	4.7
Limb extremities	273		1.5	3.3	1.8
Total	11,285	28	10.8	6.8	2.8

**Figure 3:**
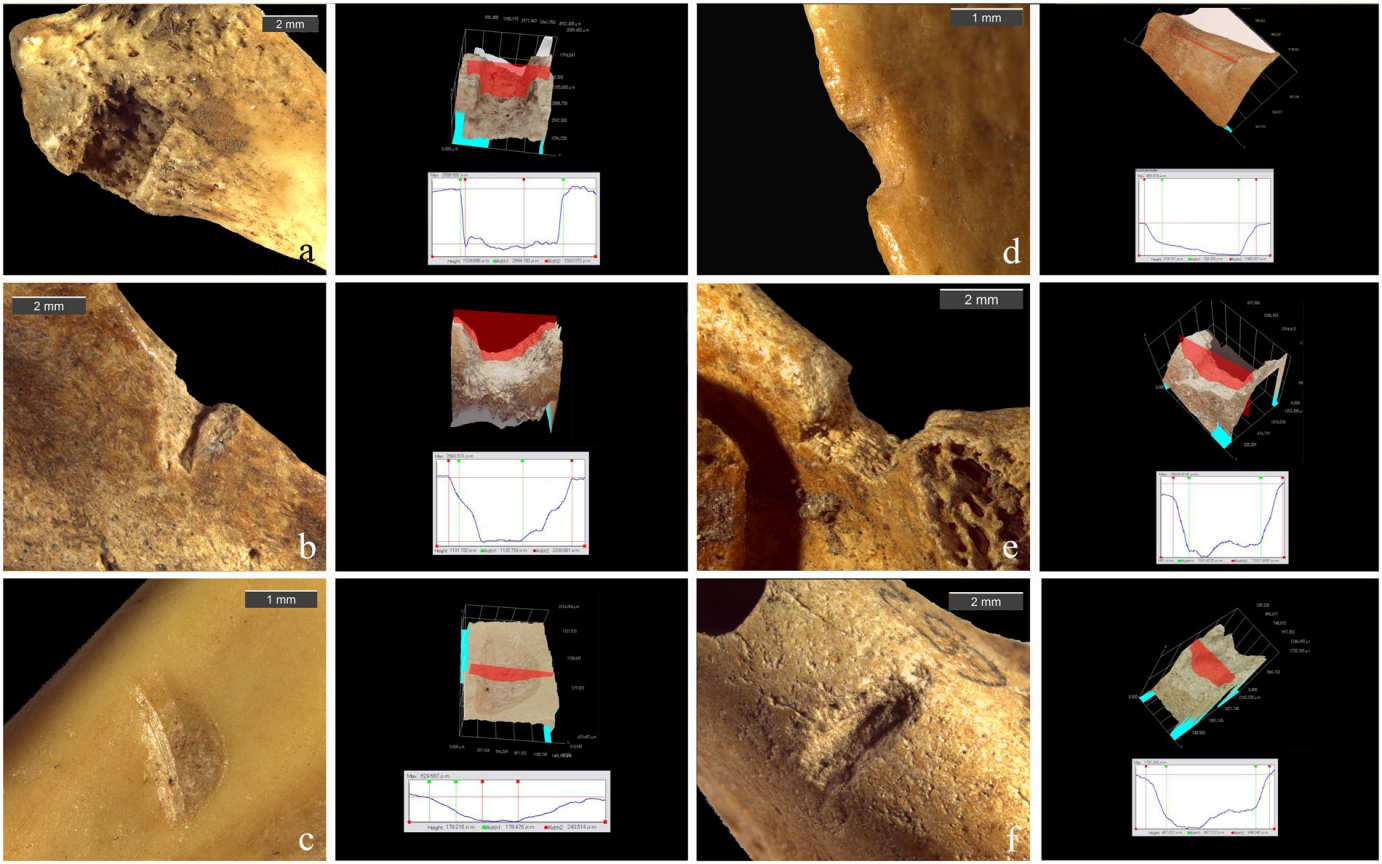
3D cross sections and stereomicroscope images of archaeological drag marks on marmot bones from Pradis Cave: (**a**) radius; (**b**) emimandible; (**c**) radius; (**d**) humerus; (**e**) coxal; (**f**) femur.

The analysis of bone surface modifications led us to the identification of several possible PIMs, morphologically comparable to drag and puncture marks obtained through the ballistic experimentation on coypu carcasses (Fig. 3). Drags were mostly located on the animals upper limbs (n = 15), especially on radius and ulna, and less frequently on femur (n = 5), coxal (n = 3) and mandible (n = 2) (Table 2 and Supplementary Table S14). Some possible drags on ribs have been evaluated but then excluded from the final counts because of the impossibility of a 3D validation. Drag profiles appear fully consistent with the experimental ones (Fig. 3 and Supplementary Fig. S15), even with the variability in conservation of the bone surfaces. A minor occurrence of flaking in respect to experimental drags and the occasional rounding of lateral slopes are in fact both related to post-depositional alterations, especially abrasion and weathering (stage 1; after Andrews^123^). Flaking is, however, visible on 24% of the cases (n = 6), whereas cracking and feathering are absent. As already suggested in a previous work^108^, this evidence confirms that a moderate post-depositional alteration of bone surfaces - in the form of roots etching, corrosion, manganese staining, trampling marks, corrosion and weathering - does not compromise the feasibility of the diagnostic method.

**Figure 4:**
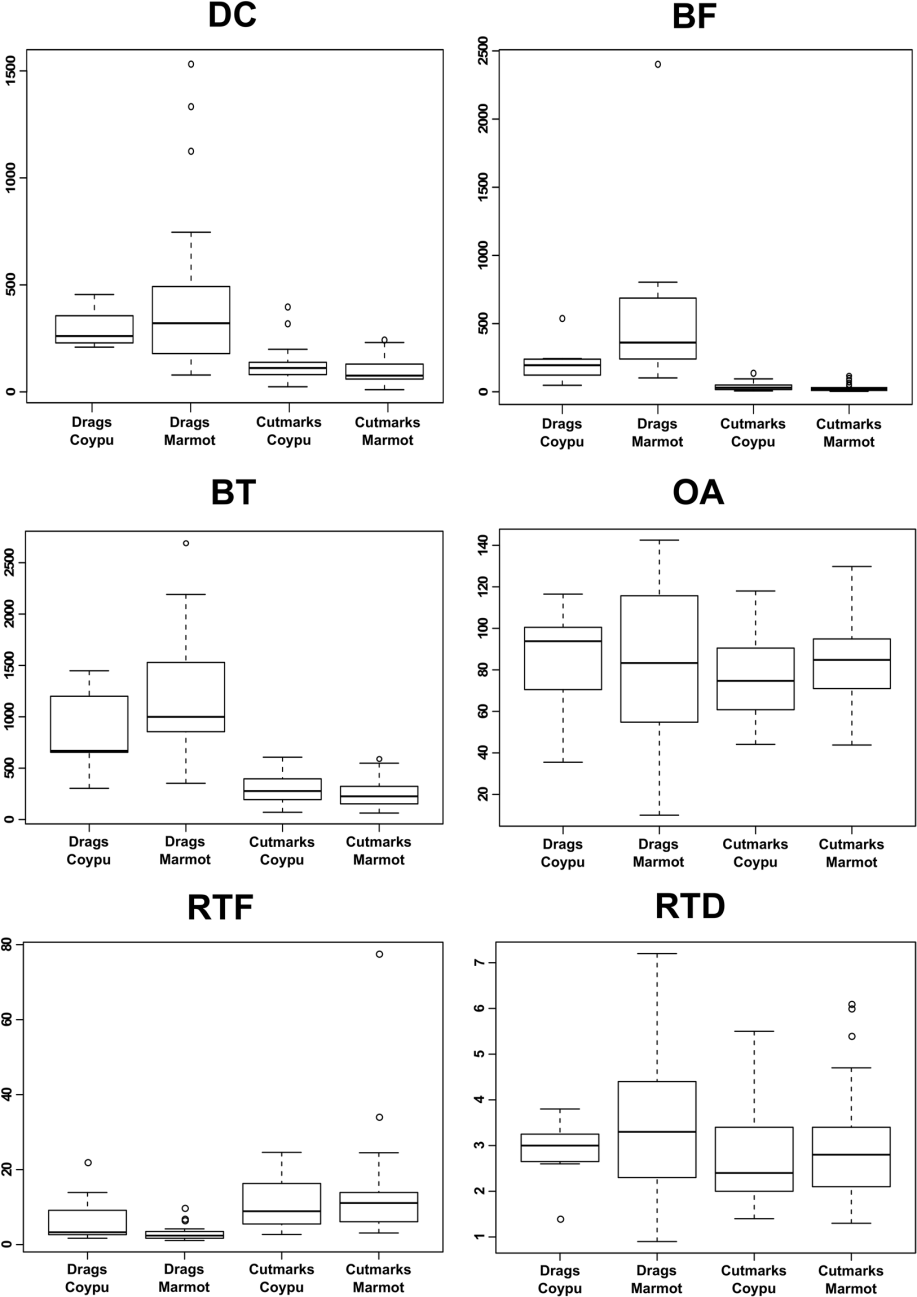
Distribution of values of different parameters measured on the analysed sample. DC: Depth of Cut; BF: Breadth at the Floor of the cut; BT: Breadth at the Top of the cut; OA: Opening Angle; RTF: Ratio between the breadth at the Top and the breadth at the Floor of the cut; RTD: Ratio between the breadth at the Top and the Depth. Experimental Drags on Coypu: n = 7; archaeological Drags on Marmot: n = 21; experimental Cut marks on Coypu: n = 35; archaeological Cut marks on Marmot: n = 37.

3D measurements, acquired on 21 of 25 specimens because of integrity issues, state that several drag morphometric parameters - such as depth of cut, breadth at the top, breadth at the floor of the cut and RTF index - are consistent with the features of experimental drags and significantly different from that of cut marks, both archaeological and experimental (Fig. 4 and Supplementary Table S16).

**Figure 5:**
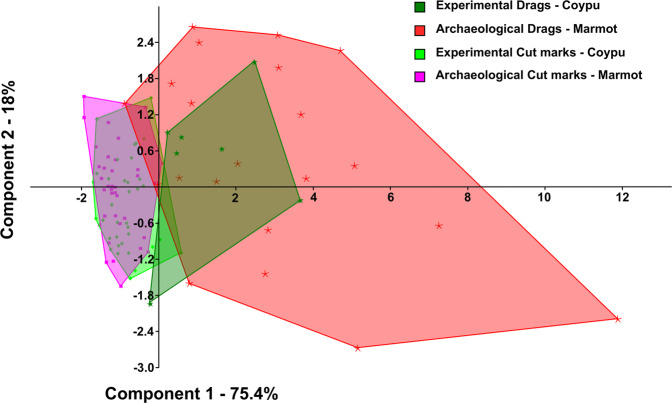
Outcome of a Principal Component Analysis carried out on the considered experimental and archaeological samples of drag marks (for sample details see the methods section). Parameters used for the test are: DC, BF, BT, SD, GD and OA (Supplementary Table S17 online).

Lastly, a Principal Component Analysis carried out on experimental and archaeological items considering all the metric parameters, confirmed the interpretation of 21 marks as impact drags with only two uncertain specimens (Fig. 5 and Supplementary Table S17). PC1, which accounts for 75.4% of variability, is mainly related to the size of marks: the greater the values are, the wider and deeper the marks, but the narrower the opening angle. PC2, which accounts for 18% of variability, is related to the shape of marks: the greater the values are, the more V-shaped and the shallower the cross-sections. Pradis archaeological drags are clearly far from the variability of cut marks and fall inside the pattern of experimental PIMs. Their position in this scatter plot is mainly influenced by their depth, breadth at the top, breadth at the floor and opening angle, confirming data presented in the boxplots (Fig. 4).

**Figure 6:**
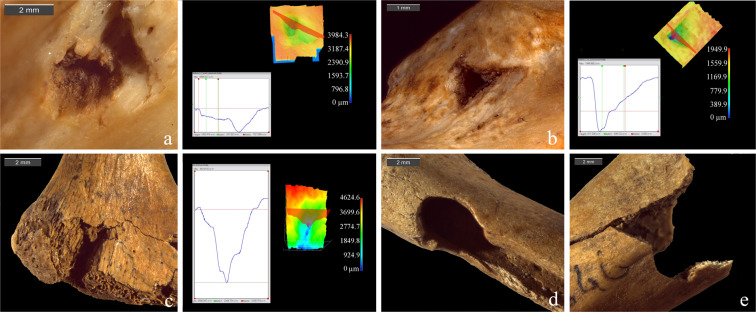
3D cross sections and stereomicroscope images of experimental punctures on mouflons (**a**, **b**) and archaeological punctures on marmot bones from Pradis Cave: (**c**) coxal; (**d**) and (**e**) femur.

Three archaeological punctures have been morphologically recognised on the base of the polygonal outline, even if two of them are associated with a fracture. They are located on a coxal and two femurs (Fig. 6). Because of integrity issues, only one was acquired in 3D but it was not possible to record all the measurements essential for a statistical comparison with experimental specimens. Nevertheless, its high depth of cavity (2,680 μm) and its sharp profile characterised by steep slopes, resemble impact punctures produced experimentally and deviate from carnivore pits and corrosion cavities^107^.

## Discussion

This study yielded three main methodological outcomes. Firstly, relatively few puncture marks were produced compared to a very high number of impact fractures and drags. This confirms that a bone dimension and thickness affects its resistance to projectile impacts, which in turn influences the representativeness of PIM classes. Impact fractures were indistinguishable from other kinds of fractures, making drag marks the most relevant category for the identification of PIMs on small game zooarchaeological remains. Secondly, the consistency of drags morphometric data coming from the two experiments revealed that drag features are not influenced by bone size and thickness, confirming the validity and feasibility of the method. A similar result has been achieved by other studies which tested the influence exerted by prey size and bone dimensions in the morphology of cut marks^124^. Lastly, archaeological drags on ribs were proven as unreliable because of the possible overlapping of their morphometric features with cut marks, making them hard to distinguish from one another.

Discussing elements useful in drag recognition, several features were confirmed as relevant for a first qualitative assessment: the location of the mark; the presence of flaking and/or cracking as secondary features; and the occurrence of lithic fragments embedded into the groove. The new experimental data confirmed the critical importance of the occurrence of lithic fragments embedded into the groove, as already assumed in a previous publication^108^ where we supported O’Driscoll and Thompson’s hypothesis^58^ that only a considerable amount of force could plunge lithic fragments into bones. Although we did not find any small lithic fragments embedded in Pradis archaeological drags, supporting evidence comes from a similar context, the Madgalenian site of La Colomb (Vercors, France). This cave, interpreted as a camp specialised in marmot hunting, yielded a marmot shoulder blade with lithic fragments fixed in two possible hunting lesions^121^.

Our study also confirms the effectiveness of 3D digital microanalysis in PIMs recognition. The collection of reproducible morphometrical parameters that can be processed through statistics, is indeed critical for the validation of the above qualitative assumptions. Even if some features - such as the depth of cut, breadth at the top, breadth at the floor and RTF index - emerged as more relevant than others in the application of this method on small mammal faunistic assemblages, the overall reliability of the diagnostic framework has been successfully tested. Moreover, we verified that a moderate post-depositional alteration of bone surfaces does not compromise the feasibility of the method on archaeological specimens.

Zooarchaeological and taphonomical analyses demonstrated that the marmot assemblage from Pradis Cave was primarily accumulated by Late Glacial hunter-gatherers. The dominance of such small prey is uncommon for Late Glacial sites of north-eastern Italy and is probably connected to the location and function of the rock shelter. The presence of at least 637 marmot individuals suggests a well-developed capture strategy and efficient hunting gear. The use of several techniques to hunt large numbers of small prey - including bows and arrows, throwing weapons, nets, snares or traps - has been reported by ethnographic and historical sources^34,125–128^. Stone-tipped projectiles seem to have been used almost exclusively on “large” game, with a few exceptions in North America and Alaska^129,130^. In light of this varied scenario, different authors questioned which capture technology could have been employed in order to make small game’s exploitation economically profitable, especially if compared to ungulates hunting^7,16,19–29,34–36,39^. As for Pradis Cave, our analysis connected marmot hunting to the use of the bow-arrow delivery system and lithic tipped projectiles, excluding the development of sophisticated acquisition techniques formerly unknown. The hypothesis that the bow and arrow was widespread in Europe during the Upper Palaeolithic has been proposed by many scholars for different chrono-cultural contexts^131–137^. Moreover, the existence of Late Epigravettian composite lithic projectiles - with a backed point hafted as piercing element and backed (bi)truncated bladelets functioning as lateral cutting elements - has been frequently proposed on the basis of backed tool’s design, use-wear analysis and the characteristics of lithic fragments found embedded in archaeological PIMs^108,133,138,139^. The Pradis data is further supported by a marmot predation using lithic tipped arrows uncovered at the La Colomb Cave site. The hunting scenario reconstructed by Tomé and Chaix^121^, wherein the animal was hit while sitting on two legs, strongly resembles the Pradis data which suggest a prevalence in predation of marmots in alert position, on the basis of experimental and archaeological PIMs location (Supplementary Fig. S18). Marmot at Pradis Cave was not an occasional prey but its exploitation appears fully incorporated in the Late Epigravettian socio-economic system. Mass harvesting was conducted without investment in new technology but maximising the energy return with a recurrent and optimised exploitation of carcasses on the site. Taphonomic analysis testified to the complete processing of the animals as sources of meat, marrow, high quality fur and possibly grease. The high percentage of defleshing marks associated to the relative low number of burned remains, led us to hypothesise the massive transportation of meat away from the site for a deferred consumption. The fur was probably removed for utilisation elsewhere. Lastly, there is evidence of bone manufacturing both for functional and symbolic purposes^118^. Data about seasonality of human visits are scarce and limited until now to marmot age classes considerations. Since 19 individuals (considering only bones affected by cut marks) have been classified as younger than 4 months, we could infer a predation between August and October, just before marmot winter hibernation (birth period: May-June; exit from the den: June-July^140^). Although this occupational pattern needs more verification (a tooth thin section analysis is ongoing), it suggests a predation during the most economically profitable period. A similar assumption has been proposed for Clusantin Cave nearby Pradis Cave^119,120^ and other marmot hunting sites dated to Late Glacial and located in the western Alpine region^21,121^. Several sites between Vercors mountains and southern Jura showed a strong connection between their altitudinal location and paleoenvironmental data, suggesting the existence of a mobility strategy specifically linked to the exploitation of high altitude resources during the season which provides the maximum energetic return^21,121^.

During the first part of the Late Glacial interstadial when Late Epigravettian human visits occurred, Pradis Cave and the close Clusantin Cave were both located below the upper limit of the tree line^141^ in an open forest environment characterised by an ecotonal zone between the woods and the alpine prairie^141^. We can thus suppose that such ecological conditions sustained large marmot colonies, well adapted to the rocky habitat along the abrupt slopes surrounding the Pradis plateau^119,120^. Therefore, the recurrent human visits in that area focusing on marmot mass exploitation could be the result of multiple factors integrating both prey availability and human choice, in the framework of the progressive human colonisation of mid-altitude territories that had been previously abandoned during the Late Glacial Maximum^142,143^. The specialisation of these sites in marmot hunting fits well in the logistical mobility system assumed for Late Epigravettian hunter-gatherers of north-eastern Italy, structured on sites that were functionally complementary to each other and located at different altitudes^144^. The continual return of humans to that area was certainly based on the local availability of this specific resource and we could not exclude that hunting with bow and arrow led humans to carry out a sort of resource management, selecting mostly adult individuals and avoiding juvenile preys, in order to allow marmot colonies to reproduce and thrive over time.

## Conclusions

This work presents new data obtained through a ballistic experiment on small-sized mammals. It also compares the results to those previously obtained on medium-sized animals, in order to bolster the diagnostic criteria useful in PIMs identification with specific reference to small game. As main methodological outcome, drag marks were recognised as the most relevant category for the identification of bone hunting lesions on small game zooarchaeological remains. Moreover, the consistency of drags morphometric data coming from the two experiments revealed that drag features are not influenced by bone size and thickness, confirming the validity and feasibility of this diagnostic method on small-sized animals.

The results presented in this work provide not only specific evidence for the identification of hunting traumas caused by Late Epigravettian projectiles, but also comparative data useful for taphonomic analyses of bones from other Late Glacial cultural contexts, especially those characterised by backed tools as projectile implements.

As already discussed, the reconstruction of hunting strategies and technology is critical to evaluate the contribution of different animal resources in human foraging efficiency. This is particularly significant in reference to small-sized mammals, whose economic role in human foraging strategies is generally underestimated. Being hunting injuries reliable indicators of the agents of bone accumulation and prey capture modalities, we thus propose to make the identification of PIMs a standard practice in zooarchaeological studies.

## Methods

### Ballistic experimentation on small-sized mammals

Coypu (*Myocastor coypus*) is a small-sized mammal very similar to alpine marmot (*Marmota marmota*) in weight and size (about 3–10 kg and 40–70 cm in head-and-body length). As such, the experiment involved 8 fresh coypu carcasses shot by 90 Late Epigravettian arrows equipped with backed points and bladelets made of flint (Supplementary Fig. S2). The backed points (length: 29 > 49 mm, mean 37.5 mm; width: 4 > 10 mm, mean 7.4 mm; thickness: 2 > 5.5 mm, mean 4.0 mm) were mounted in a lateral groove at the top of the shaft. 60 arrows had their points associated with two backed bi-truncated bladelets (length: 13 > 31 mm, mean 22 mm; width: 5 > 8 mm, mean 6.4 mm; thickness: 1.5 > 5 mm, mean 3.0 mm): of these, half were fixed parallel to the shaft as a prolongation of the point’s lateral cutting edge, while the other half was arranged obliquely like barbs (Supplementary Fig. S2). A composite glue made of resin, ochre and beewax was used to secure each microlith to the shaft. The animals were suppressed within two hours from the start of the experimental session by officers of the Italian State Forestry Corps within the provincial (Provincia Autonoma di Trento) control policies of infesting animals as part of routine pest control. The carcasses, complete and not subjected to any treatment, were loosely suspended from a wooden bracket in a lifelike position, as detailed in Supplementary Fig. S19. The archers were all experimental practitioners with extensive experience in public demonstrations and traditional bow competitions since the 1990s. The bows were made from *Maclura pomifera* and *Fraxinus* sp. wood with a poundage of 43 lb and 38 lb respectively. The archers shot a maximum of 20 arrows on a single carcass from a distance between five and seven meters; each arrow was shot only once. The carcasses were skinned with metal knives at the end of every shooting session, then grouped in net containers and macerated in water. After three months, they were taken out and gently washed in water without any chemical substances. In addition, two more coypu carcasses were directly butchered using stone flakes (made of the same flint used to manufacture the projectile implements) in order to collect experimental cut marks on small mammal bones.

### Analytical methods

Taxonomic and skeletal identifications were carried out on the basis of the complete alpine fauna reference collection of MUSE-Science Museum of Trento. Marmot NMI was calculated on the number of left emimandible while the evaluation of age classes was based on dental use-wear and eruption stages from Couturier collection of the Natural History Museum of Grenoble, already analysed by Fournier^145^ and Gay^146^. Taphonomic analyses were conducted at MUSE-Science Museum of Trento using a stereomicroscope (Leica M 165 C with magnification from 0.75 to 125×). The identification of the surface alterations and the distinction of different taphonomic agents were carried out with reference to well-established literature^41–57^. The descriptive criteria and the terminology used for PIMs, follows O’Driscoll and Thompson^58^ as already discussed in Duches *et al*.^107^.

The 3D analysis of bone surfaces was conducted at the University of Siena using a Hirox Digital Microscope KH-7700, with MXG-10C body, OL-140II lens and AD-10S Directional Lighting Adapter^91,147–149^. Each drag was analysed through the acquisition of different metrical parameters, according to criteria already published (Fig. 4^107^) and using one cross-section per mark in its median part (Supplementary Fig. S1). In addition, other measurements were calculated as the RTD index (the ratio between breadth at the top and depth of cut) and the RTF index (the ratio between the breadth at the top and the breadth at the floor of the cut). Profiles and measures acquired on archaeological specimens were compared to drags produced experimentally by Late Epigravettian projectiles on coypus (results in this report) and mouflon carcasses (n = 7)^107^. Of the total number of drags identified in the archaeological assemblage, only a part could be analysed through 3D microscopy due to the incompleteness of some marks because of either the total removal of a bone edge or the absence of a continuous floor.

Moreover, the values were compared to 123 experimental cut marks produced with unretouched flakes: 22 were inflicted on cattle autopodials^91^, 66 on cat carcasses^148^ and 35 on coypu carcasses (this report). Regarding the tooth marks compared to experimental drags, they consisted of 58 present-day scores found on ungulate carcasses collected in the field and were attributable to small wild carnivores and dogs^107^. All the measurements considered in this paper have been directly acquired by two of the authors (FB and JC), using the software directly installed in the digital microscope. They also processed the data in the various statistical tests using the PAST software^150^.

## Supplementary information

Supplementary Information.

## Data Availability

All data generated or analysed during this study are included in this published article (and its online Supplementary Information files).
