# Supplementary material for: Experimental and archaeological data for the identification of projectile impact marks on small-sized mammals
Source: Sci Rep. 2020 Jun 4;10:9092. doi: 10.1038/s41598-020-66044-3 (PMC7272403; doi:10.1038/s41598-020-66044-3)

Supplementary Information

Experimental and archaeological data for the identification of projectile impact marks on small-sized mammals

Rossella Duches*
MUSE - Museo delle Scienze (MUSE)
Corso del Lavoro e della Scienza 3, IT 38123, Trento - Italy
rossella.duches@muse.it

Nicola Nannini
MUSE - Museo delle Scienze (MUSE)
Corso del Lavoro e della Scienza 3, IT 38123, Trento - Italy

Alex Fontana
MUSE - Museo delle Scienze (MUSE)
Corso del Lavoro e della Scienza 3, IT 38123, Trento - Italy

Francesco Boschin
Università degli Studi di Siena
Dipartimento di Scienze Fisiche, della Terra e dell'Ambiente, UR Preistoria e Antropologia
Via Laterina 8, IT 53100, Siena - Italy

Jacopo Crezzini
Università degli Studi di Siena
Dipartimento di Scienze Fisiche, della Terra e dell'Ambiente, UR Preistoria e Antropologia
Via Laterina 8, IT 53100, Siena - Italy

Marco Peresani
Università degli Studi di Ferrara
Dipartimento di Studi Umanistici, Sezione di Scienze Preistoriche e Antropologiche, Corso Ercole I d' Este 32, IT 44121, Ferrara - Italy

Supplementary text

Late Pleistocene adaptations in the Alps

The peopling of north-eastern Italy after the Last Glacial Maximum was a gradual process marked by the progressive colonization of new territories that had been previously abandoned, following changes of vegetation and animal distribution[1-2]. Late Epigravettian penetration, firstly limited to valley floors and high plateaus around 500 m asl[3-4], reaches mid-altitude territories during the second part of the Late Glacial interstadial with the full development of a logistical occupation network at the ecotone between coniferous woods and alpine prairies[5]. This organisation corresponds to a seasonal mobility strategy that sometimes included sites that are functionally complementary to each other and located at different altitudes[5]. Technological analysis of the lithic industries from the most relevant sites has highlighted a progressive simplification of the lithic production system throughout the Late Glacial interstadial[6-7] associated with the persistence of standardised lithic backed tools used as projectile implements[8]. The existence of specific retouched forms, characterised by distinct metric and morphological parameters, reveals as many mental templates developed by the Epigravettians in response to specific functional requirements and constraints[8]. These constraints fit within the conception of the bow-arrow delivery system, whose generalised use in the Epipaleolithic complexes of Europe has been hypothesised by many scholars[9-13]. Functional analyses carried out on Late Epigravettian industries of north-eastern Italy[4, 14-16] confirmed the exclusive use of lithic backed tools as elements in hunting projectiles.

Pradis Caves

The Pradis Caves are three rock shelters located at 560 m a.s.l. in the Pradis Plateau, on the eastern side of the Carnic Prealps in the Friuli Venezia Giulia region (Italy). This orographic unit of 850 km^2 is characterised by an uneven landscape with steep gradients and valleys from 400 to 800 m deep, bordered by mountains reaching 2000-2300 m.

The Pradis Plateau covers an area of over 6 km^2 at an elevation of 530-590 m, wedged between Pala Mount (1,231 m), Rossa Mount (1,369 m) and Ciaurlec Mount (1,148), and is bordered to the south by the gorge of the Cosa stream that flows across the sub-alpine hills and onto the upper Friulian plain. The plateau, between the plain and the Prealps, was of strategic importance for access to the mountain range and the upper basin of the Tagliamento River. The hydrographic network, partly characterised by impermeable substrate (Clauzetto Flysch), developed tight and deep ravines (Torrente Cosa and Rio Secco), with numerous natural shelters and caves excavated in carbonate formations. In a similar way to those opening at the bottom of collapsed sink-holes, some of the

cavities were filled with Pleistocene sediments. Among these are Pradis Caves, used during the Mousterian and the Late Glacial Epigravettian[17-19], Rio Secco Caves with Mousterian and Gravettian levels[20-23] and Clusantin Cave[4, 24]. The latter opens at short distance from Pradis Caves, on the eastern side of a wide sink-hole partially filled by alluvial terrace deposits.
Despite the abundance of paleontological and archaeological materials found, the three shelters (Riparo I, II and III) of the Pradis Caves (or Grotte Verdi di Pradis) were radically transformed in a tourist place of worship and refreshment, with the consequent loss of most of the information sealed in the sediments. The deposits spared were investigated in 1970 and 1971 under the direction of a palaeontologist, Giorgio Bartolomei (University of Ferrara)[17-18]. All the sediment was wet sieved with very fine meshes, as the primary aim of the investigation was to collect micromammals teeth and bones for bio-chronological studies.
Of the three shelters, that of major interest was the Riparo I by the presence of Pleistocene deposits two meters thick, but limited to a surface of a little over three square meters backed to the wall of the bottom and connected to the rock vault. The stratigraphic succession is articulated in a set of units mainly formed by the accumulation of cryoclastic gravel with variable content of silts and guano levels. The lower part is a thick layer of gravel with clayey matrix: levels 13 to 7, stripped by artificial levels, contained almost exclusively cave bear bones and some rare Mousterian lithic artefacts. Above, a level of guano (6) is covered with low silt gravel (5) and gravel with higher amount of silt (layers 4 to 2). Finally, layers 2 and 1 (the latter being split into 1a and 1b) yielded abundant bones of mammals, especially marmots and a deciduous human tooth currently under study. Two cut-marked marmot bones – an emimandible and a scapula – selected from the faunal sample of level 1a, were AMS radiocarbon dated between 13.9 and 12.6 Cal BP as reported in Supplementary Table S9.
The lithic artefacts from levels 1 and 2 can be considered fairly homogeneous, referred to the Late Epigravettian; the flint is alloctonous, probably from the basin of the Venetian Prealps[18].
Along with lithic artefacts, some bone tools have also been preserved: two marmot clavicles with incised notches and two bone points (layer 1a)[19].
The faunal association records few large cervids (moose, red deer) and caprids (ibex and chamois) with cut marks and percussion marks that testify Epigravettian hunters targeted a variety of herbivores on the plateau and surroundings (see Supplementary Table S11). However, the most targeted game was the alpine marmot (*Marmota marmota*), as reported in the main text. Marmot remains show an evident standardised butchering scheme that allows the reconstruction of various processing phases[25]. Pradis Caves alongside the neighbouring Clusantin cave[24], describe the Pradis

plateau like a unique area of specialised hunting and exploitation of this rodent during the Late Upper Palaeolithic.

1. Naudinot, N., Tomasso, A., Tozzi, C. & Peresani, M. Changes in mobility patterns as a factor of 14C date density variation in the Late Epigravettian of Northern Italy and Southeastern France. *Journal of Archaeological Science*, **52**, 578-590 (2014).
2. Ravazzi, C., Peresani, M., Pini, R. & Vescovi, E. Il Tardoglaciale nelle Alpi e in Pianura Padana: evoluzione stratigrafica, storia della vegetazione e del popolamento antropico. Il Quaternario, *Italian Journal of Quaternary Sciences*, **20(2)**, 163-184 (2007).
3. Aimar, A. *et al.* Les Abris Villabruna dans la Vallée du Cismon. *Preistoria Alpina*, **28**, 227-254 (1992).
4. Peresani, M., Duches, R., Miolo, R., Romandini, M. & Ziggiotti, S. Small Specialized Hunting Sites and their Role in Epigravettian Subsistence Strategies. A Case Study in Northern Italy in *Hunting Camps in Prehistory. Current Archaeological Approaches* (eds Bon, F., Costamagno, S. & Valdeyron, N.). *P@lethnology*, **3**, 251-266 (2011).
5. Bertola, S. *et al.* L'Epigravettiano recente nell'area prealpina e alpina orientale in *L'Italia tra 15.000 e 10.000 anni fa, Cosmopolitismo e regionalità nel Tardoglaciale* (ed Martini, F.) *Studi di Archeologia Preistorica*, **5** (Millenni, Museo Fiorentino di Preistoria "Paolo Graziosi") 39-94 (2007).
6. Montoya, C. Evolution des concepts de productions lithiques et artistiques à l'Epigravettien récent: analyses de collections des Préalpes de la Vénétie et des Préalpes du sud françaises in *Il Tardiglaciale in Italia. Lavori in corso* (ed Mussi, M.). *Brit Archaeol Rep*, **1859**, 43-55 (2008).
7. Montoya, C. & Peresani, M. Premiers éléments de diachronie dans l'Epigravettien récent des Préalpes de la Vénétie in D'un monde à l'autre. Les systèmes lithiques pendant le Tardiglaciaire autour de la Méditerranée nord-occidentale (eds Bracco, J.P. & Montoya, C.). *Mémoire de la Societé Préhistorique Française*, **XL**, 123-138 (2005).
8. Duches, R., Peresani, M. & Pasetti, P. Success of a flexible behavior. Considerations on the manufacture of Late Epigravettian lithic projectile implements according to experimental tests. *Archaeological and Anthropological Sciences*, **10(7)**, 1617-1643 (2018).
9. Caspar, J.P. & De Bie, M. Preparing for the hunt in the Late Paleolithic camp at Rekem, Belgium. *J Field Archaeol*, **23**, 437-460 (1996).
10. Cattelain, P. Hunting during the Upper Paleolithic: bow, spearthrower, or both in *Projectile Technology* (ed Knecht, H.) 213-240 (Plenum Press: New York, 1997).

11. Pelegrin, J. Les techniques de débitage laminaire au Tardiglaciaire: critères de diagnose et quelques réflexions in *L'Europe centrale et septentrionale au Tardiglaciaire* (eds Valentin, B., Bodu, P. & Christensen, M.). *Mémoires du Musée de Prèhistoire d'Ile-de-France*, **7**, 73-86 (2000).
12. Plisson, H. Examen tracéologique des pointes aziliennes du Bois-Ragot in *La grotte du Bois-Ragot à Gouex (Vienne). Magdalénien et Azilien. Essais sur les hommes et leur environnement* (eds Chollet, A. & Dujardin, V.). *Mémoire de la Société Préhistorique Française*, **XXXVIII**, 183-189 (2005).
13. Valentin, B. *Jalons pour une paléohistoire des derniers chasseurs (XIVe-VIe millénaire avant J.-C.)* (Publications de la Sorbonne, Cahiers Archéologiques de Paris 1: Paris, 2008).
14. Lemorini, C., Rossetti, P., Cusinato, A., Dalmeri, G., Hrozny Kompatscher, N.M. & Kompastcher, K. L'analisi delle tracce d'uso e l'elaborazione spaziale: il riconoscimento di un'area specializzata nel sito epigravettiano di Riparo Dalmeri, livelli 26b e 26c (Trento). *Preistoria Alpina*, **41**, 171-197 (2006).
15. Ziggiotti, S. Studio funzionale delle armature microlitiche dei siti del Piancavallo (Pordenone). *Bollettino della Società Naturalisti "Silvia Zenari"*, **30**, 37-511 (2006).
16. Ziggiotti, S. Strategie di caccia degli ultimi epigravettiani. Lo studio funzionale delle armature litiche di Riparo La Cogola, livello 19. *Preistoria Alpina*, **43**, 13-24 (2008).
17. Bartolomei, G., Broglio, A. & Palma di Cesnola, A. Chronostratigraphie et écologie de l'Epigravettien en Italie in *La fin des temps glaciaires en Europe* (ed De Sonneville-Bordes, D.). *Colloques Internationaux du C.N.R.S.*, **271**, 297-324 (1979).
18. Corai, P. Le più antiche culture preistoriche della Ladinia (Paleolitico e Mesolitico). *Ladinia,* **IV**, 183-218 (1980).
19. Gurioli, F., Bartolomei, G., Nannini, N., Peresani, M. & Romandini, M. Deux clavicules de marmotte epigravettiennes incisées, provenant des Grottes Verdi de Pradis (Alpes Italiennes). *Paléo*, **22**, 311-318 (2011).
20. Peresani, M. *et al.,*. New evidence for the Neanderthal demise and earliest gravettian occurrences at Rio Secco cave, Italy. *J. F. Archaeol,* **39**, 401-416 (2014).
21. Talamo, S. *et al.,*. Detecting human presence at the border of the northeastern Italian Pre-Alps. 14C dating at Rio Secco Cave as expression of the first Gravettian and the late Mousterian in the northern Adriatic region. *Plos One,* **9(4)**, e95376 (2014).
22. Romandini, M. *et al.,*. Convergent evidence of eagle talons used by Late Neanderthals in Europe: a further assessment on symbolism. *Plos One,* **9(7)**, e101278 (2014).

23. Romandini, M., Terlato, G., Nannini, N., Tagliacozzo, A., Benazzi, S. & Peresani, M.. Humans and Bears a Neanderthal tale. Reconstructing uncommon behaviors from zooarchaeological evidence in southern Europe. *Journal of Archaeological Science*, **90**, 71-91 (2018).
24. Romandini, M., Peresani, M., Gurioli, F. & Sala, B. *Marmota marmota*, the most common prey species at Grotta del Clusantin: Insights from an unusual case-study in the Italian Alps. *Quaternary International*, **252,** 184-194 (2012).
25. Nannini, N. *Tra archeozoologia, paleobalistica e antropologia. Lettura degli impatti delle armi da getto epigravettiane su resti faunistici nel Tardoglaciale dell'Italia nord-orientale* (Phd thesis: University of Ferrara, 2018).

Supplementary Figure S1: Measurements taken on drag marks and cut marks profiles. DC: depth of cut; BT: breadth at the top of the cut; BF: breadth at the floor of the cut; GD/SD: greatest and smallest distances from the middle of the floor to the edges; OA: opening angle

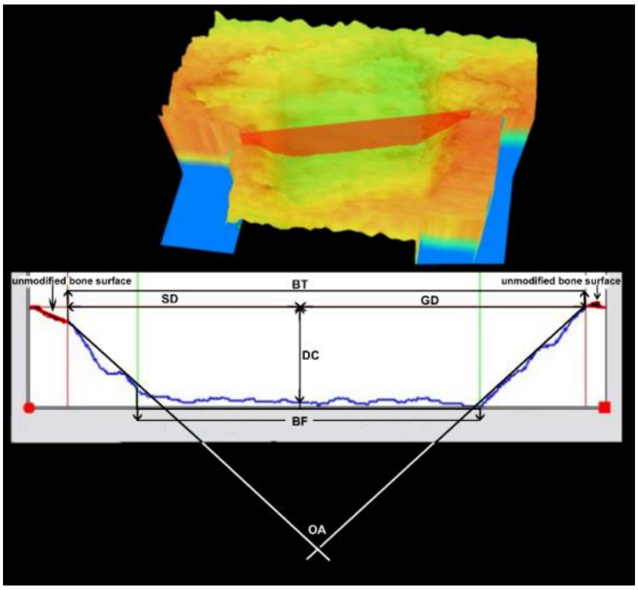

Supplementary Figure S2: Different arrangements of Late Epigravettian projectiles used in the experiments; a) one single backed point mounted obliquely on the shaft in a lateral groove and two backed (bi)truncated bladelets fixed parallel to the shaft; b) one single backed point mounted obliquely on the shaft in a lateral groove and two backed (bi)truncated bladelets fixed oblique as barbs; c) one single backed point mounted obliquely on the shaft in a lateral groove.

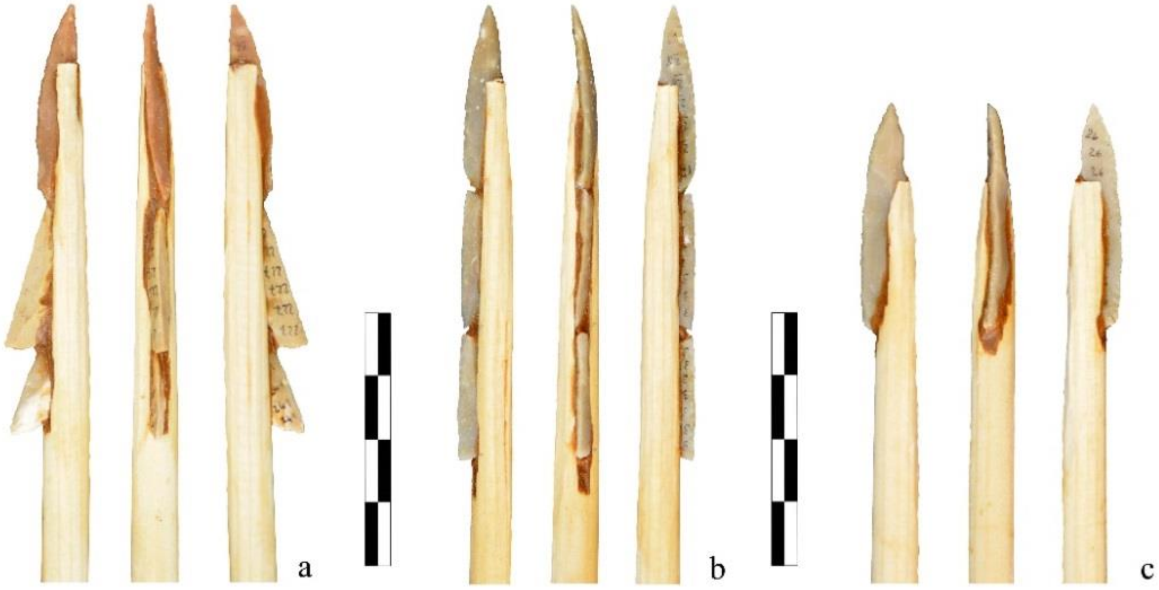

Supplementary Table S3: Comparison of the ballistic results obtained through the two experimentations.

	**N. shots**	**N. hits**	***% hits***	**N. PIMs**	***% PIMs***
**Medium-sized game**	160	143	*89.4*	70	*49.0*
**Small-sized game**	90	77	*85.6*	66	*85.7*

Supplementary Figure S4: Location of the experimental PIMs produced on coypu carcasses subdivided on the base of the animal position: a) standing on two legs facing the archers; b) standing on two legs positioned backwards with respect to the archers; c) on four legs with left side facing the archers. The drawing of the *Marmota marmota* skeleton (a, b) was made by Cédric Beauval and Carine Tomé Carpentier while the drawing of the *Castor fiber* skeleton (c) was made by Michel Coutureau. Both were taken from ArcheoZoo.org (available at: https://www.archeozoo.org/archeozootheque/picture/2892-marmota_marmota/category/144-rongeurs_langen_rodents_lang_langes_roedores_lang_;https://www.archeozoo.org/archeozootheque/picture/2889-castor_fiber/category/144-rongeurs_langen_rodents_lang_langes_roedores_lang_ under the following licence https://creativecommons.org/licenses/by-sa/4.0/) and then modified by us with the addition of the location of the experimental PIMs. The castor skeleton was further modified by us to be more similar to a marmot one (we slightly changed the body proportion – shortening the tail and the hind leg – and replaced the cranium with the marmot one).

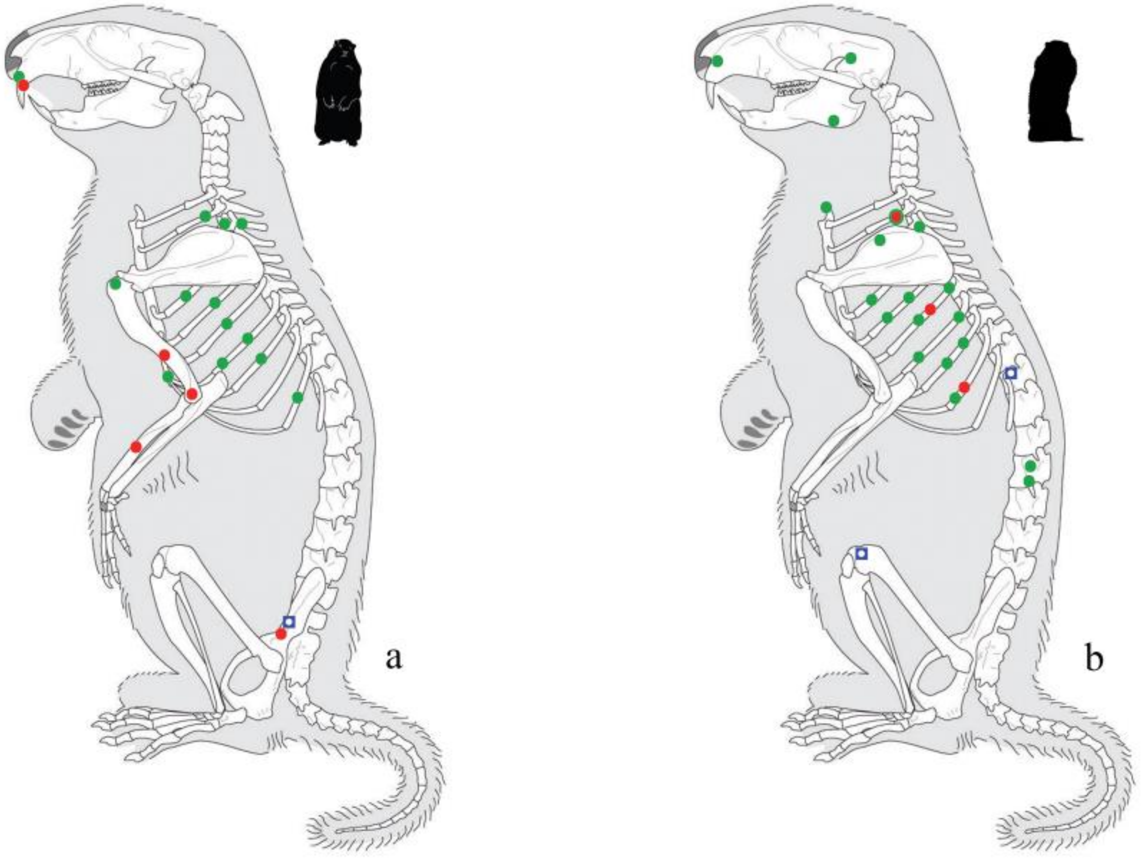

© 2005 ArcheoZoo.org / Cédric Beauval, Carine Tomé Carpentier
After : Morel, Ph. & Muller, W. Hauterive-Champréveyres 11 : Un campement magdalénien au bord du lac de Neuchâtel : étude archéozoologique (secteur 1). *Archéologie Neuchâteloise* **23**, 74-82 (1997)

Former categories
- drag
- fracture
- puncture

Sub-categories
- puncture/fracture
- drag/fracture
- stone embedded

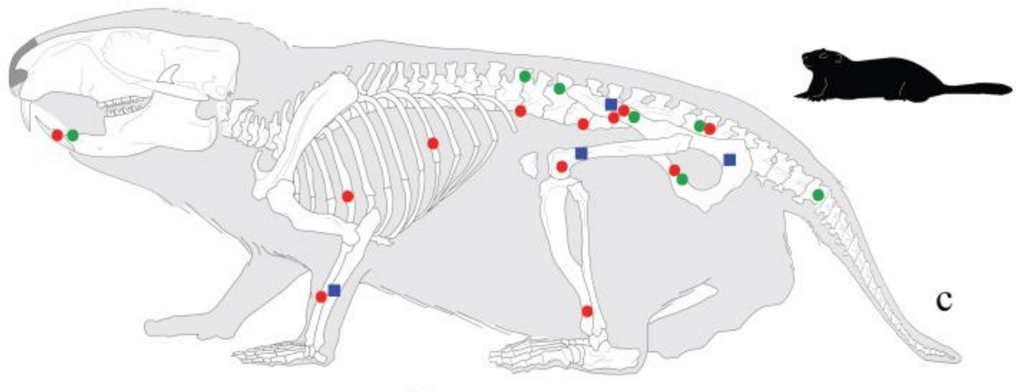

© 2003 ArcheoZoo.org / Michel Coutureau (Inrap)
After: Pales, L. & Garcia M. A. *Atlas ostéologique pour servir à l'identification des Mammifères du Quaternaire - Herbivores.* (Paris: CNRS, 1981)

Supplementary Figure S5: 3D cross sections and stereomicroscope images of experimental drag marks on coypu bones made by Late Epigravettian lithic projectiles: a) radius, b) incisor, c) and d) coxal.

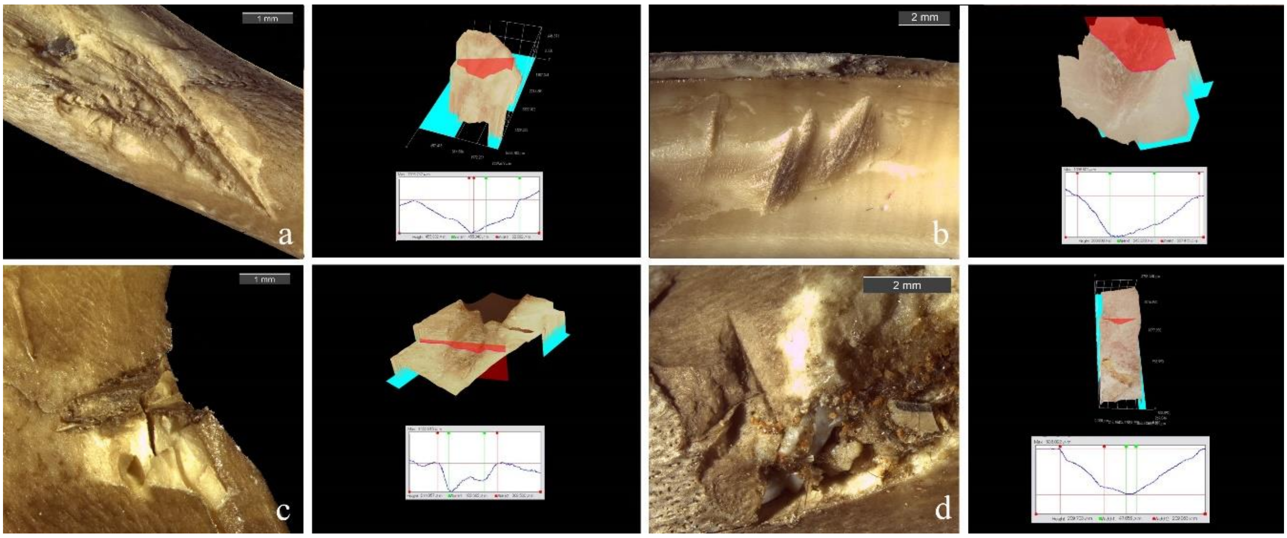

Supplementary Figure S6: Stereomicroscope images of experimental PIMs on coypu bones made by Late Epigravettian lithic projectiles: a, b) puncture with stone embedded on femur; c, d) puncture with stone embedded on coxal; e, f) puncture with stone embedded on thoracic vertebra; g) fracture on caudal vertebra; h) fracture on lumbar vertebra; i) fracture on humerus; l) fracture on coxal; m) puncture/fracture on femur; n) puncture/fracture on coxal.

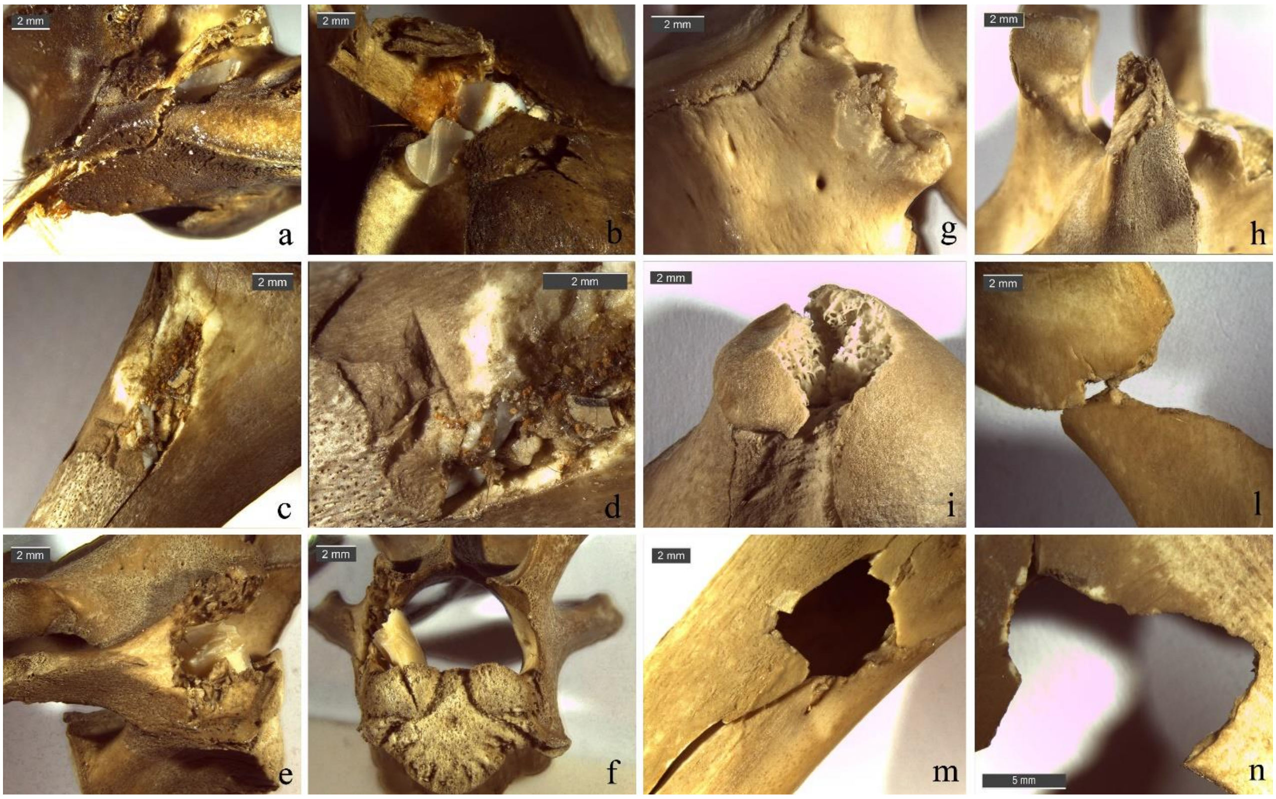

Supplementary Figure S7: 3D cross sections of experimental cut marks on coypu bones: a) emimandible; b) clavicle; c) humerus; d) coxal; e) fibula; f) caudal vertebra.

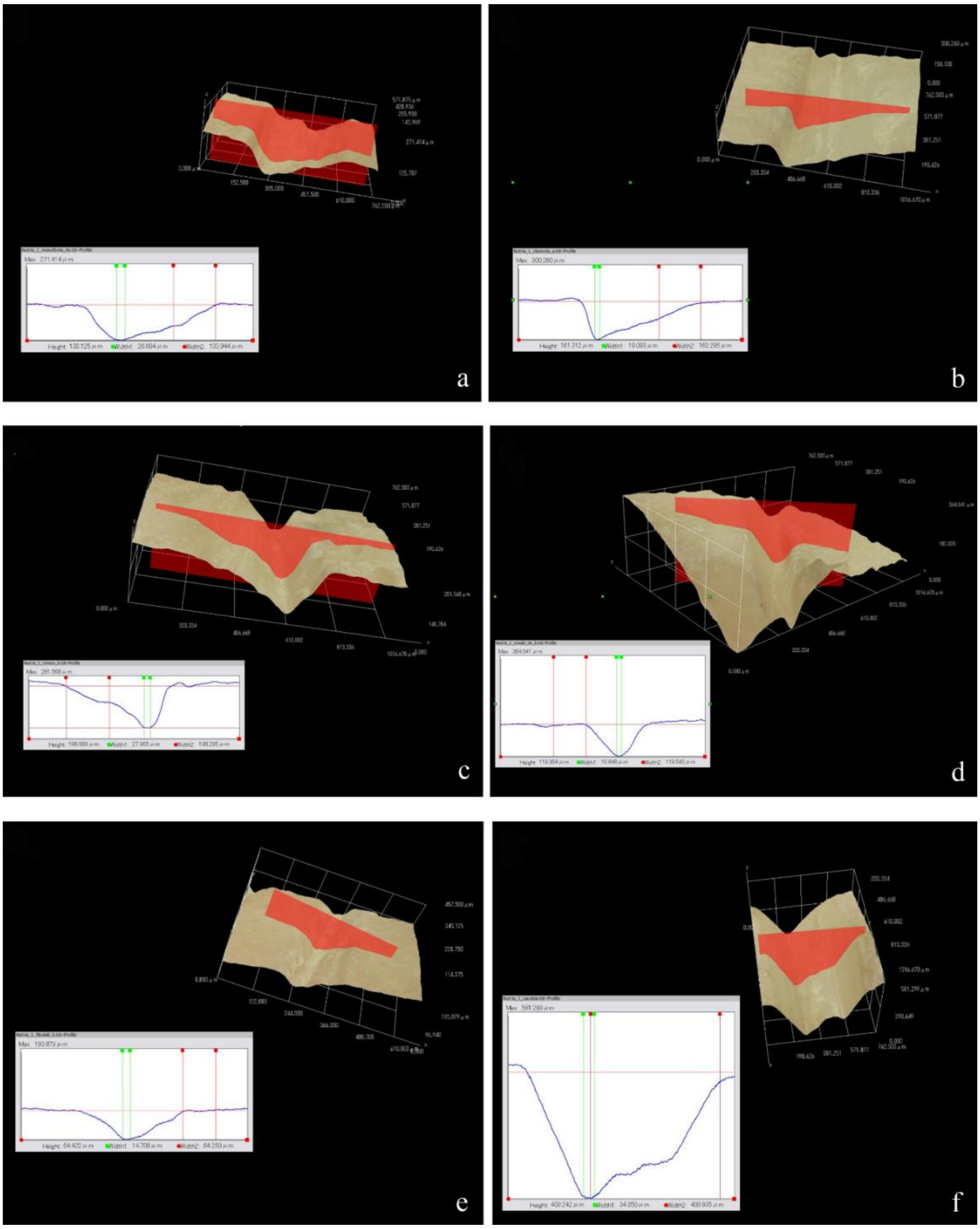

Supplementary Table S8: Raw data of experimental drags on coypus, experimental cut marks on coypus, experimental drags on ungulates, experimental cut marks on ungulates, experimental cut marks on cats and actual carnivore tooth scores. DC: depth of cut; BF: breadth at the floor of the cut; BT: breadth at the top of the cut; GD: greatest distance from the middle of the floor to the edges; SD: smallest distance from the middle of the floor to the edges; OA: opening angle.

Group	DC	BF	BT	GD	SD	OA	Name	Bone element
Experimental Drags - Coypu	442,9	548	1448	791	657	56,2	OST00001_001 Nutria1Giorno1Omerodx	Humerus
Experimental Drags - Coypu	455	62	1366	712	654	116,5	OST00001_002 Nutria1Giorno1Radiodx	Radius
Experimental Drags - Coypu	268,7	235	1034	642	392	84,8	OST00006_003 Nutria1Giorno3Radio	Radius
Experimental Drags - Coypu	209	47,6	660,8	334	326,8	100	OST00005_001 Nutria3Giorno2Coxale	Innominate
Experimental Drags - Coypu	260,9	243,3	668,8	364,6	304,2	101	OST00006_001 Nutria 1 Incisivo	Incisor
Experimental Drags - Coypu	243	194,8	648,2	392,4	255,8	93,8	OST00006_001 Nutria_1_Incisivo	Incisor
Experimental Drags - Coypu	214,8	182,7	303,6	160,2	143,4	35,5	OST00007_001 Nutria 2 T72 coxale	Innominate
Experimental Drags - Coypu (Ribs only)	211	212	372	205	167	58	OST00003_001 Nutria1Giorno2Costa	Rib
Experimental Drags - Coypu (Ribs only)	147	121,7	319	166	153	85	OST00006_002 Nutria1Giorno3Costa	Rib
Experimental Drags - Coypu (Ribs only)	262,5	159,1	427,9	285,9	142	64,7	OST00004_001 Nutria2Giorno2Costa	Rib
Experimental Drags - Coypu (Ribs only)	95,9	75,9	200,6	111	89,6	70,4	OST00004_002 Nutria2Giorno2Costa	Rib
Experimental Cut marks - Coypu	403	34	601	409	192	51,9	OST00009_012 Nutria1Caudale	Caudal vertebra
Experimental Cut marks - Coypu	161,3	19	466,5	401	65,5	80,3	OST00009_002_a Nutria1Clavicola_a	Clavicle
Experimental Cut marks - Coypu	24	13,7	84	56,3	27,7	91	OST00009_002_b Nutria1Clavicola_b	Clavicle
Experimental Cut marks - Coypu	48,9	22,4	122,7	92,8	29,9	73	OST00009_002_c Nutria1Clavicola_c	Clavicle
Experimental Cut marks - Coypu	136	21,9	290	150	140	66,6	OST00009_007_a Nutria1CoxaleDx_a	Innominate
Experimental Cut marks - Coypu	119,3	16,8	209,4	121	88,4	70,9	OST00009_007_b Nutria1CoxaleDx_b	Innominate
Experimental Cut marks - Coypu	63,9	94,7	255,6	140,5	115,1	97,6	OST00009_009_a Nutria1FibulaA_a	Fibula
Experimental Cut marks - Coypu	127	58,8	398,5	253,5	145	80,7	OST00009_009_c Nutria1FibulaA_b	Fibula
Experimental Cut marks - Coypu	95,3	41,9	196,6	135,5	61,1	64,4	OST00009_009_c Nutria1FibulaA_c	Fibula
Experimental Cut marks - Coypu	113	61,2	274	162	112	68,9	OST00009_010_a Nutria1FibulaB_a	Fibula
Experimental Cut marks - Coypu	64,4	14,7	197,7	109,5	88,2	90	OST00009_010_b Nutria1FibulaB_b	Fibula
Experimental Cut marks - Coypu	45,5	6,3	153,2	92,2	61	118	OST00009_010_c Nutria1FibulaB_c	Fibula
Experimental Cut marks - Coypu	133	26,6	437	302	135	100	OST00009_001 Nutria1Mandibola	Mandible
Experimental Cut marks - Coypu	111,5	47	391	302	89	94,7	OST00009_003 Nutria1OmeroA	Humerus
Experimental Cut marks - Coypu	199	27,9	469	370	99	53,7	OST00009_004 Nutria1OmeroB	Humerus
Experimental Cut marks - Coypu	91,5	145,9	485,1	334,1	151	101,2	OST00009_005_a Nutria1Radiodx_a	Radius
Experimental Cut marks - Coypu	84,5	21,1	142,75	57,6	85,15	59,9	OST00009_005_b Nutria1Radiodx_b	Radius
Experimental Cut marks - Coypu	44	14,3	106,7	68,8	37,9	94,3	OST00009_005_c Nutria1Radiodx_c	Radius
Experimental Cut marks - Coypu	142,8	16	296,1	150,8	145,3	87,7	OST00009_005_d Nutria1Radiodx_d	Radius
Experimental Cut marks - Coypu	109,5	35,6	235	150	85	60,6	OST00009_006 Nutria1Radiosin	Radius
Experimental Cut marks - Coypu	161,1	83,2	393,9	228,1	165,8	86,6	OST00009_008_a Nutria1Tibiasin_a	Tibia
Experimental Cut marks - Coypu	113,7	77,3	410,5	270,7	139,8	100,7	OST00009_008_b Nutria1Tibiasin_b	Tibia
Experimental Cut marks - Coypu	117,5	21,2	308	216,6	91,4	62,4	OST00010_002_a Nutria2Costa2_a	Rib
Experimental Cut marks - Coypu	138,8	15,5	271	177,7	93,3	44,1	OST00010_002_b Nutria2Costa2_b	Rib
Experimental Cut marks - Coypu	160,1	29,8	293	134,3	158,7	47,6	OST00010_003_a Nutria2CostA3_a	Rib

Experimental Cut marks - Coypu	106,3	19,9	179,1	125,2	53,9	79,5	OST00010_003_b Nutria2CostA3_b	Rib
Experimental Cut marks - Coypu	76,8	52	359,9	274,6	85,3	83,8	OST00010_004 Nutria2CostaDorsale	Rib
Experimental Cut marks - Coypu	110	62,5	606,5	347,1	259,4	115,4	OST00010_005_a Nutria2FemoreCaputDx_a	Femur
Experimental Cut marks - Coypu	137,6	21,8	193,4	124,8	68,6	61	OST00010_005_b Nutria2FemoreCaputDx_b	Femur
Experimental Cut marks - Coypu	95,9	37,2	194,2	125,8	68,4	56,8	OST00010_005_c Nutria2FemoreTrocantereDx_a	Femur
Experimental Cut marks - Coypu	325	28,7	464,5	326,5	138	60	OST00010_005_d Nutria2FemoreTrocantereDx_b	Femur
Experimental Cut marks - Coypu	96,97	76,14	328,9	227,3	101,6	81,1	OST00010_005_e Nutria2FemoreTrocantereDx_c	Femur
Experimental Cut marks - Coypu	41,8	10,5	183,4	161,5	21,9	62,4	OST00010_001_a Nutria2ParietaleSin_a	Skull
Experimental Cut marks - Coypu	32,9	12,8	70,5	53,9	16,6	74,7	OST00010_001_b Nutria2ParietaleSin_b	Skull
Experimental Cut marks - Coypu	144,9	15,8	277,1	213,5	63,6	55,7	OST00009_011 Nutria1Sacro1	Sacrum
Experimental Drags - Ungulates	266	55	338	200	138	87,66	Mufl_III_15_tacca	Mandible
Experimental Drags - Ungulates	606	1890	2868	1525	1343	94,5	Mufl_II_16_tacca_grande	Radius
Experimental Drags - Ungulates	170	148	472	301,7	170,3	80,76	Mufl_III_23_tacca	Radius
Experimental Drags - Ungulates	319	224	765	390	375	86,58	Mufl_III_26_tacca	Coxal
Experimental Drags - Ungulates	1000	227	1525	896,8	628,2	71,66	Mufl_IV_38_tacca	Rib
Experimental Drags - Ungulates	1112	186	713	429	284	65,84	Mufl_I_5_tacca	Vertebra
Experimental Drags - Ungulates	262	383	851	470	381	79,25	Mufl_III_34_tacca_er	Rib
Experimental Cut Marks - Ungulates	34,6	22	205,5	115	90,5	129,0	Fal1_Str4b2	Phalanx 1
Experimental Cut Marks - Ungulates	49,1	19,6	234	124	110	120	Faldx_Str2a1	Phalanx 1
Experimental Cut Marks - Ungulates	24,5	8,5	109	77	32	109	Faldx_Str12a1	Phalanx 1
Experimental Cut Marks - Ungulates	91,3	28,1	285,4	197	88,4	132	Faldx_Str3a2	Phalanx 1
Experimental Cut Marks - Ungulates	12,7	16,3	193,1	110	83,1	149	Fal1_Str2b3	Phalanx 1
Experimental Cut Marks - Ungulates	37	13,3	285	224	61	135	Faldx_Str11a2	Phalanx 1
Experimental Cut Marks - Ungulates	20,1	21,6	185	109	76	138	Fal1_Str1d5	Phalanx 1
Experimental Cut Marks - Ungulates	24,9	17,8	114	77	37	131	Faldx_Str1a3	Phalanx 1
Experimental Cut Marks - Ungulates	71,8	21,2	202	102	100	119	Faldx_Str5a3	Phalanx 1
Experimental Cut Marks - Ungulates	37,3	44	277	152	125	144	Faldx_Str6a2	Phalanx 1
Experimental Cut Marks - Ungulates	47,5	24,5	218	120	98	111	Faldx_Str7a1	Phalanx 1
Experimental Cut Marks - Ungulates	23	45,5	192,5	104	88,5	132	Faldx_Str8a3	Phalanx 1
Experimental Cut Marks - Ungulates	28,5	8,6	115	82	33	110	Faldx_Str13a1	Phalanx 1
Experimental Cut Marks - Ungulates	23,1	5,6	142	110	32	114	Mc_Str1a3	Phalanx 1
Experimental Cut Marks - Ungulates	30,7	10,1	120,6	74	46,6	99	Mc_Str2a1	Metacarpal
Experimental Cut Marks - Ungulates	56	13	292,4	160	132,4	99	Fal2_Str3d2	Phalanx 2
Experimental Cut Marks - Ungulates	87,6	20	348	183	165	106	Faldx_Str4a2	Phalanx 1
Experimental Cut Marks - Ungulates	58,8	49	291,8	205	86,8	126	Faldx_Str9a1	Phalanx 1
Experimental Cut Marks - Ungulates	54,2	17	266,5	167	99,5	118	Fal1_Str5a3	Phalanx 1
Experimental Cut Marks - Ungulates	31,9	32,2	196	146	50	116	Fal1_Str6a2	Phalanx 1
Experimental Cut Marks - Ungulates	17,7	133	190,2	106	84,2	143	Faldx_Str10a1	Phalanx 1
Experimental Cut Marks - Ungulates	114,3	70	435	297	138	90	Mc_Str3a2	Metacarpal
Experimental Cut Marks - Cat	75,3	23,2	119	68,9	50,1	67,8	Gatto1mc1_1	Metacarpal
Experimental Cut Marks - Cat	117,4	22,6	193	136,3	56,7	58,9	Gatto1mc1_3	Metacarpal
Experimental Cut Marks - Cat	38,9	26,2	107,2	75,8	31,4	76	Gatto1mc1_4	Metacarpal
Experimental Cut Marks - Cat	127	23,7	324,6	227,9	96,7	100	Gatto1mc1_5	Metacarpal
Experimental Cut Marks - Cat	89	22	136,23	78	58,23	58,2	Gatto1mc1_6	Metacarpal
Experimental Cut Marks - Cat	87,6	38,3	299,3	170,9	128,4	98	Gatto2fal1_1	Phalanx 1
Experimental Cut Marks - Cat	110	24,4	443	3,2	439,8	125	Gatto2fal1_2	Phalanx 1
Experimental Cut Marks - Cat	188	76,8	368	253	115	52,9	Gatto2fal2_1	Phalanx 2

Experimental Cut Marks - Cat	179,6	25,6	378,8	314,8	64	76,6	Gatto2fal2_2	Phalanx 2
Experimental Cut Marks - Cat	135	36,7	256,6	130	126,6	95	Gatto3fal1_1	Phalanx 1
Experimental Cut Marks - Cat	78,7	21,9	266,5	175,3	91,2	107,4	Gatto3fal1_2	Phalanx 1
Experimental Cut Marks - Cat	87,5	20,2	229,8	154,4	75,4	88,6	Gatto3fal1_3	Phalanx 1
Experimental Cut Marks - Cat	73,7	18,8	257	188,6	68,4	79,2	Gatto3fal1_4	Phalanx 1
Experimental Cut Marks - Cat	15,3	17,8	98	49	49	134,9	Gatto3fal1II_1	Phalanx 1
Experimental Cut Marks - Cat	75,6	26,3	313	167	146	110	Gatto3fal2_1	Phalanx 2
Experimental Cut Marks - Cat	56	89	246	158,2	87,8	111	Gatto3fal2_2	Phalanx 2
Experimental Cut Marks - Cat	96	28	184	107	77	117	Gatto3fal2_4	Phalanx 2
Experimental Cut Marks - Cat	25,4	76,5	201	127,3	73,7	131,2	Gatto3fal2_7	Phalanx 2
Experimental Cut Marks - Cat	35,5	42,5	143,5	78,5	65	111,1	Gatto3fal2_8	Phalanx 2
Experimental Cut Marks - Cat	30,3	14,2	117,6	72,5	45,1	123,7	Gatto1crdx1	Skull
Experimental Cut Marks - Cat	42	15,4	170	94,3	75,7	115,5	Gatto1crdx2	Skull
Experimental Cut Marks - Cat	28,4	6,6	111,2	71,8	39,4	107,6	Gatto1crsin1	Skull
Experimental Cut Marks - Cat	34,9	6,6	116,1	58,2	57,9	111	Gatto1crsin3	Skull
Experimental Cut Marks - Cat	30,5	19,9	198	133	65	128	Gatto1manddx1	Mandible
Experimental Cut Marks - Cat	12,4	14,6	109,5	62,9	46,6	149,2	Gatto1mandsin1	Mandible
Experimental Cut Marks - Cat	36,4	47,9	114	63,4	50,6	89,1	Gatto1tib10	Tibia
Experimental Cut Marks - Cat	25,6	20,9	191,9	150,1	41,8	119,2	Gatto1tib3	Tibia
Experimental Cut Marks - Cat	9,2	17	46	26	20	99,9	Gatto1tib5	Tibia
Experimental Cut Marks - Cat	33,4	10,1	74,8	54	20,8	64,8	Gatto1tib6	Tibia
Experimental Cut Marks - Cat	36,7	6,8	71,8	40,8	31	85,4	Gatto1tib7	Tibia
Experimental Cut Marks - Cat	42	10,3	110,6	75,4	35,2	91,2	Gatto1tib9	Tibia
Experimental Cut Marks - Cat	43,4	13	110	72	38	83	Gatto2manddx1	Mandible
Experimental Cut Marks - Cat	34,2	24	111,3	66,2	45,1	99	Gatto2manddx2	Mandible
Experimental Cut Marks - Cat	83	14	183	135,9	47,1	71	Gatto2manddx3	Mandible
Experimental Cut Marks - Cat	44,8	12	125	92,9	32,1	78,6	Gatto2manddx4	Mandible
Experimental Cut Marks - Cat	80	21,7	304	239	65	70	Gatto2mandsin1	Mandible
Experimental Cut Marks - Cat	9	24,9	65,5	35	30,5	122,2	Gatto2mandsin2	Mandible
Experimental Cut Marks - Cat	54	14,3	64,1	33	31,1	129,1	Gatto2mandsin3	Mandible
Experimental Cut Marks - Cat	18,7	78,2	143,6	78,9	64,7	119,3	Gatto3calc1	Calcaneum
Experimental Cut Marks - Cat	58,4	10,9	165,4	137,7	27,7	79,7	Gatto3calc2	Calcaneum
Experimental Cut Marks - Cat	41,8	11,9	130,9	83,3	47,6	99,3	Gatto3calc3	Calcaneum
Experimental Cut Marks - Cat	13,2	12,4	88,7	62,5	26,2	137,4	Gatto3calc4	Calcaneum
Experimental Cut Marks - Cat	38	219,3	359,3	194,6	164,7	118,3	Gatto3cr1	Skull
Experimental Cut Marks - Cat	24,6	18,6	174,1	107,9	66,2	147,8	Gatto3cr2	Skull
Experimental Cut Marks - Cat	20,8	49,6	273,7	187,8	85,9	151,7	Gatto3cr3	Skull
Experimental Cut Marks - Cat	33,9	93,2	308,4	205	103,4	129,9	Gatto3cr4	Skull
Experimental Cut Marks - Cat	14	34	141,9	100,2	41,7	128	Gatto3cr5	Skull
Experimental Cut Marks - Cat	38,7	17,4	109,7	77,7	32	82	Gatto3fibsin1	Fibula
Experimental Cut Marks - Cat	47,8	29	206	139	67	108,8	Gatto3fibsin2	Fibula
Experimental Cut Marks - Cat	16,7	8	159	111,8	47,2	138	Gatto3mc1_1	Metacarpal
Experimental Cut Marks - Cat	47,7	18,5	167,7	110,7	57	89,2	Gatto3mc1_2	Metacarpal
Experimental Cut Marks - Cat	17,8	7,1	59,5	39,7	19,8	92,9	Gatto3mt1_1	Metatarsal
Experimental Cut Marks - Cat	14,8	11,7	60,9	36,7	24,2	105,9	Gatto3mt1_2	Metatarsal
Experimental Cut Marks - Cat	23,5	12,9	115,9	88,5	27,4	95,6	Gatto3mt1_3	Metatarsal
Experimental Cut Marks - Cat	20	13,3	76	42	34	106,4	Gatto3tibia1_1	Tibia

Experimental Cut Marks - Cat	47	11,5	91,7	55,6	36,1	79,9	Gatto3tibia1_3	Tibia
Experimental Cut Marks - Cat	21,6	7,5	64,3	44,7	19,6	100,2	Gatto3tibia1_5	Tibia
Experimental Cut Marks - Cat	52,4	11	126,1	73,3	52,8	86,9	Gatto3tibia1_6	Tibia
Experimental Cut Marks - Cat	15,2	10	60,4	33,4	27	120	Gatto3tibia1_7	Tibia
Experimental Cut Marks - Cat	76	17,5	210,5	123,6	86,9	75	Gatto3tibia1_8	Tibia
Experimental Cut Marks - Cat	28	26,9	68,3	34,5	33,8	69,2	Gatto3tibia1_9	Tibia
Experimental Cut Marks - Cat	22	40	95	51,8	43,2	103	Gatto3tibia2_1	Tibia
Experimental Cut Marks - Cat	32	24	134	94,2	39,8	106	Gatto3tibia2_2	Tibia
Experimental Cut Marks - Cat	7,5	11	50,8	26,8	24	129,3	Gatto3tibia2_3	Tibia
Experimental Cut Marks - Cat	28	30,6	156	86,3	69,7	120	Gatto3tibia2_5	Tibia
Experimental Cut Marks - Cat	36	9,4	151,3	97	54,3	127	Gatto3tibia2_6	Tibia
Tooth Marks	33,6	138	264	148	116	123,7	Fem1CcPr1a	Femur
Tooth Marks	64,7	271,1	350	210	140	80,8	Om1CcPr2a	Humerus
Tooth Marks	17,7	96,1	271,3	150	121,3	146	Om2CcP3a	Humerus
Tooth Marks	20,8	130,5	230	141	89	116	Om3CcPr1a	Humerus
Tooth Marks	35,2	365,1	420,2	230	190,2	91,9	Om3CcPr3a	Humerus
Tooth Marks	60,3	205,3	498	293	205	134,4	Ra1CoConf1a	Radius
Tooth Marks	62	268,2	576,8	381	195,8	141,6	RaCo5	Radius
Tooth Marks	58,4	342,2	739	404	335	151,7	RaCo10	Radius
Tooth Marks	46,3	197,6	559,4	289	270,4	148,5	251MandCO1	Mandible
Tooth Marks	87,9	154	767,1	404	363,1	146,9	251MandCO3	Mandible
Tooth Marks	74,5	362,8	760	486	274	140,68	251MandCO4	Mandible
Tooth Marks	61,4	458,37	801	400	401	152,4	251MandCO5	Mandible
Tooth Marks	56	337,2	505,1	271	234,1	126,9	251MandCO7	Mandible
Tooth Marks	66,9	426	587,4	313	274,4	126,6	251MandCO8	Mandible
Tooth Marks	76,4	273	860	436	424	144,3	251MandCO9	Mandible
Tooth Marks	66,5	359	760,4	397	363,4	147,1	251MandCO10	Mandible
Tooth Marks	73,3	290,6	507,4	286	221,4	122,2	RaCo1	Radius
Tooth Marks	71	127	450,6	345	105,6	127,6	RaCo2	Radius
Tooth Marks	38,7	187,5	379,8	234	145,8	135,5	RaCo4	Radius
Tooth Marks	28,6	16,1	141,6	88,7	52,9	118,2	RaCo6	Radius
Tooth Marks	25,5	15,1	143,6	89	54,6	115,8	RaCo7	Radius
Tooth Marks	54,7	317,8	627,4	368	259,4	148,1	RaCo9	Radius
Tooth Marks	78	186	656,7	451	205,7	129,3	251MandCO2	Mandible
Tooth Marks	45,9	43,1	407,7	229	178,7	128,2	Ra1CoConf2a	Radius
Tooth Marks	46,5	205,9	326,2	168	158,2	136,5	Om1CcPr1a	Humerus
Tooth Marks	27,2	246,2	318,9	165	153,9	127,7	Om2CcPr1a	Humerus
Tooth Marks	21,3	237,5	304,7	161	143,7	116	Om2CcP2a	Humerus
Tooth Marks	29,7	187,1	302,3	166	136,3	131,9	Om3CcPr2a	Humerus
Tooth Marks	29,1	215,4	410,6	210	200,6	148,2	MandCcdxOl1a	Mandible
Tooth Marks	21,2	95,2	277,8	155	122,8	152,7	Om2CcP3a	Humerus
Tooth Marks	62,8	458	904,5	493	411,5	146,2	Ra1CoConf3a	Radius
Tooth Marks	28,4	237,5	465	285	180	140,7	Ra1CoConf4a	Radius
Tooth Marks	50,7	161,7	658	422	236	155,7	RaCo3	Radius
Tooth Marks	72,3	454,9	849	500	349	146,6	RaCo8	Radius
Tooth Marks	190	206	840	479	361	113,5	HumFrosini9a	Humerus
Tooth Marks	53,6	136	463,6	259	204,6	143,98	HumFrosini1a	Humerus

Tooth Marks	23,9	99,3	264,2	144	120,2	144,2	HumFrosini4a	Humerus
Tooth Marks	78,2	288	622	330	292	119	HumFrosini6a	Humerus
Tooth Marks	56,5	128,8	362	235	127	119,5	HumFrosini8a	Humerus
Tooth Marks	17	114,8	331,7	215	116,7	155,6	HumFrosini2a	Humerus
Tooth Marks	38,5	228	422,4	226	196,4	151,4	HumFrosini3a	Humerus
Tooth Marks	27,1	117,4	346	186	160	155,2	HumFrosini5a	Humerus
Tooth Marks	19	393	583	318	265	160,7	HumFrosini7a	Humerus
Tooth Marks	28	105,2	335	177	158	158,4	Ti135collez9a	Tibia
Tooth Marks	45	223	377	184	193	131,7	Ti135collez4a	Tibia
Tooth Marks	120	719	1130	619	511	126,4	Ti135collez5a	Tibia
Tooth Marks	40	278	450	253	197	143,2	Ti135collez1a	Tibia
Tooth Marks	117,3	240	793	417	376	132,2	Mt135collez3a	Metatarsal
Tooth Marks	65,4	340	743	388	355	155,7	Mt135collez1a	Metatarsal
Tooth Marks	42,6	287	616,6	317	299,6	157,5	Mt135collez2a	Metatarsal
Tooth Marks	42	406	568	330	238	131,2	Mt135collez4a	Metatarsal
Tooth Marks	81,7	234	809	437	372	147,4	Mt135collez5a	Metatarsal
Tooth Marks	31,4	475	601	325	276	157,3	Ti135collez2a	Tibia
Tooth Marks	42,4	268	491	305	186	133,1	Ti135collez3a	Tibia
Tooth Marks	39,6	271	394	210	184	141,9	Ti135collez6a	Tibia
Tooth Marks	15	337,6	516	293	223	156,5	Ti135collez7a	Tibia
Tooth Marks	13,6	186,2	301	163	138	154	Ti135collez8a	Tibia
Tooth Marks	16,2	232	346	197	149	151,6	Ti135collez10a	Tibia

Supplementary Table S9: Radiocarbon ages of two marmot bones with cut marks from Pradis Caves. Dates were obtained from CEntro di DAtazione e Diagnostica (CEDAD), University of Salento; dates were calibrated using software OxCal Ver. 3.10 (Reimer PJ, et al. 2004 Radiocarbon 46:1029-1058)

**Layer**	**Laboratory number**	**Age BP**	**CAL BP 2σ range**	**Material dated**
1a	LTL13258A	12,273 ± 50	11,950-12,650 (*92.4%*)	Marmot bone with cut marks
			12,750-12,900 (*3%*)	
1a	LTL13259A	12,832 ± 50	12,950-13,950 (*95.4%*)	Marmot bone with cut marks

Supplementary Figure S10: Location of Pradis Caves in north-eastern Italy (map obtained through Google Earth Pro; https://www.google.it/intl/it/earth/) and stratigraphic sketch with highlighted the Late Epigravettian layers.

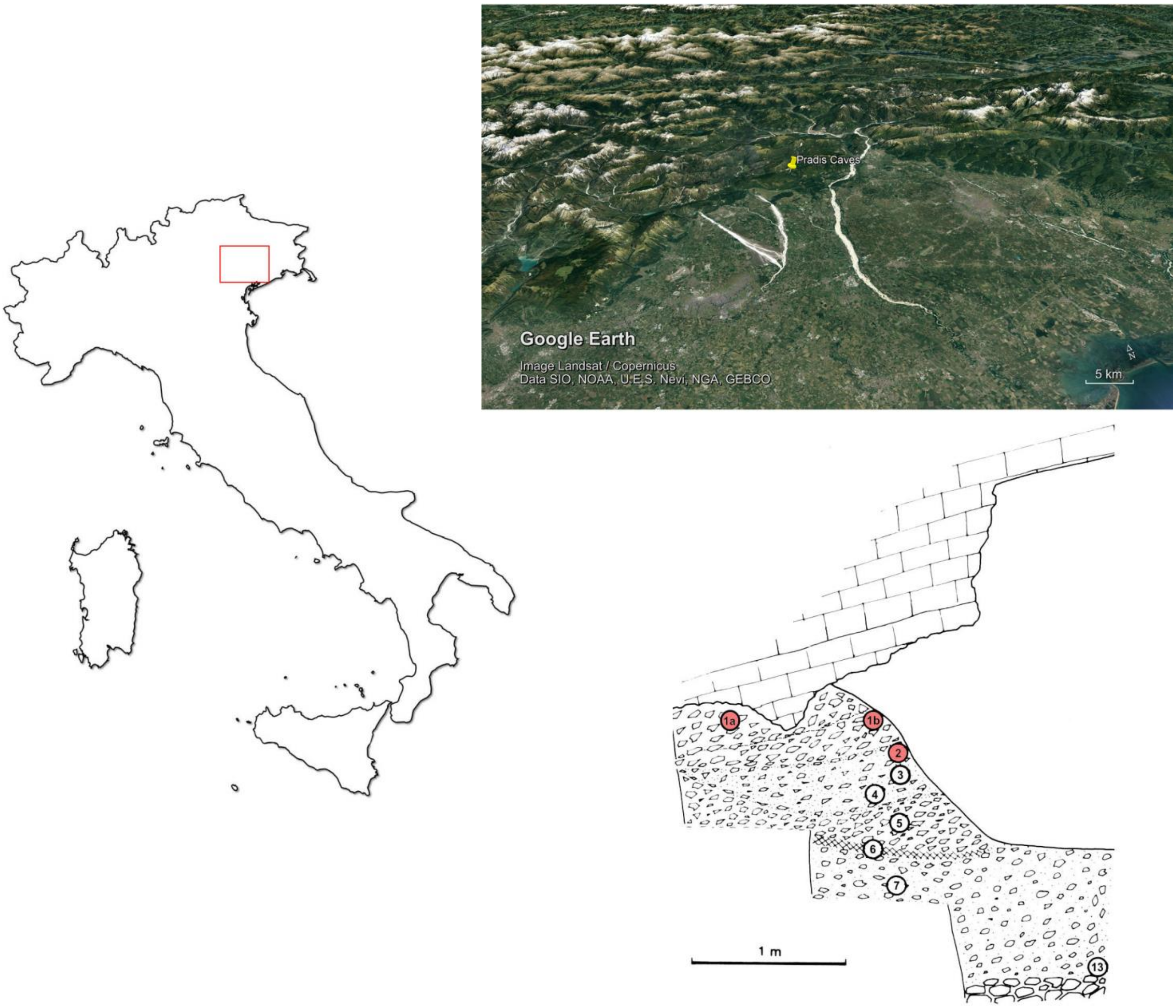

Supplementary Table S11: Faunal assemblage from Riparo I of Pradis Caves (layers 1 and 2).

Taxa	**NISP**	***NISP%***	**MNI**
Marmota marmota	11,285	*99.2*	637
Lepus sp.	9	*0.07*	2
Martes foina	1	*0.008*	1
Gulo gulo	1	*0.008*	1
Vulpes vulpes	1	*0.008*	1
Canis sp.	2	*0.01*	1
Felis sp.	1	*0.008*	1
Ursus arctos	1	*0.008*	1
Carnivora	1	*0.008*	
Capreolus capreolus	2	*0.01*	1
Cervus elaphus	9	*0.07*	4

Alces alces	17	*0.14*	3
Cervidae large size	4	*0.03*	
Rupicapra rupicapra	12	*0.1*	2
Capra ibex	3	*0.02*	2
Caprinae	1	*0.008*	1
Bos/Bison	8	*0.07*	2
Ungulata	4	*0.03*	
Unidentified large size	3		
Aves	6		
**Total**	**11,371**		**660**

Supplementary Table S12: Marmot NMI by age from Riparo I of Pradis Caves.

Months	MNI
0 - 13	106
13 - 26	125
22 - 29	72
26 - 48	2
> 36	254
**Total**	**559**

Supplementary Figure S13: 3D cross sections and stereomicroscope images of archaeological cut marks on marmot bones from Pradis Cave: a) scapula; b) humerus; c) tibia.

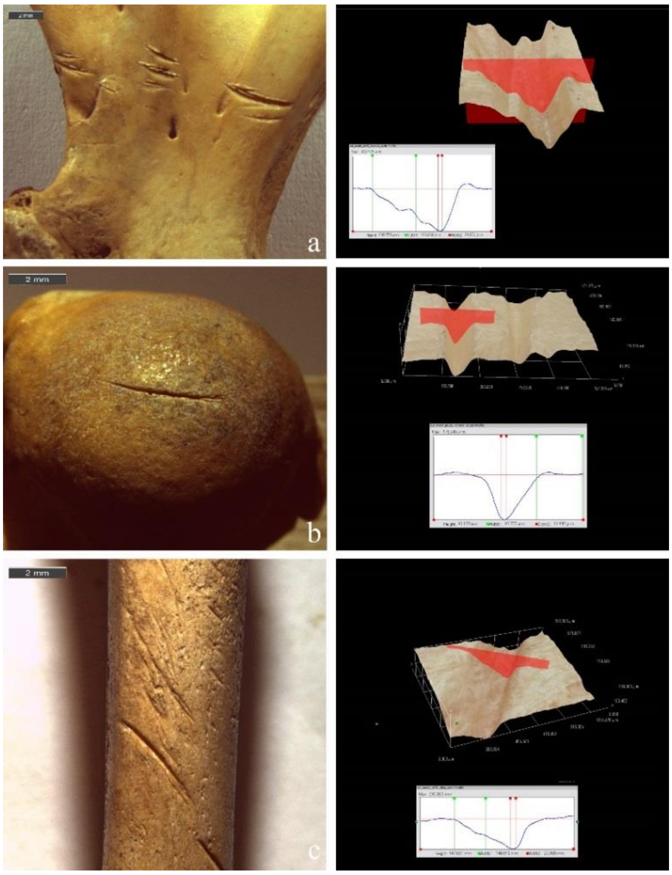

Supplementary Table S14: Number of anatomical elements of marmot assemblage and indication of PIM categories.

	**NISP**	**Drag**	**Puncture**	**Puncture/Fracture**
Cranium	1,455			
Hemimandible	1,324	2		
Tooth	1,689			
Vertebra	678			
Rib	878			
Clavicle	126			
Scapula	680			
Humerus	629	2		
Radius	569	7		
Ulna	701	6		
Radius-ulna	1			
Carpal	0			
Metacarpal	98			
Coxal	651	3	1	
Femur	535	5		2
Tibia	547			
Malleolar bone	162			
Calcaneum	54			
Astragalus	52			
Tarsal	0			
Metatarsal	183			
Metapodial	28			
I phalanx	172			
II phalanx	39			
III phalanx	34			
**Total**	**11,285**	**25**	**1**	**2**

Supplementary Figure S15: 3D cross sections and stereomicroscope images of archaeological drag marks on marmot bones from Pradis Cave: a) and b) radius; c) and d) femur; e) coxal; f) ulna.

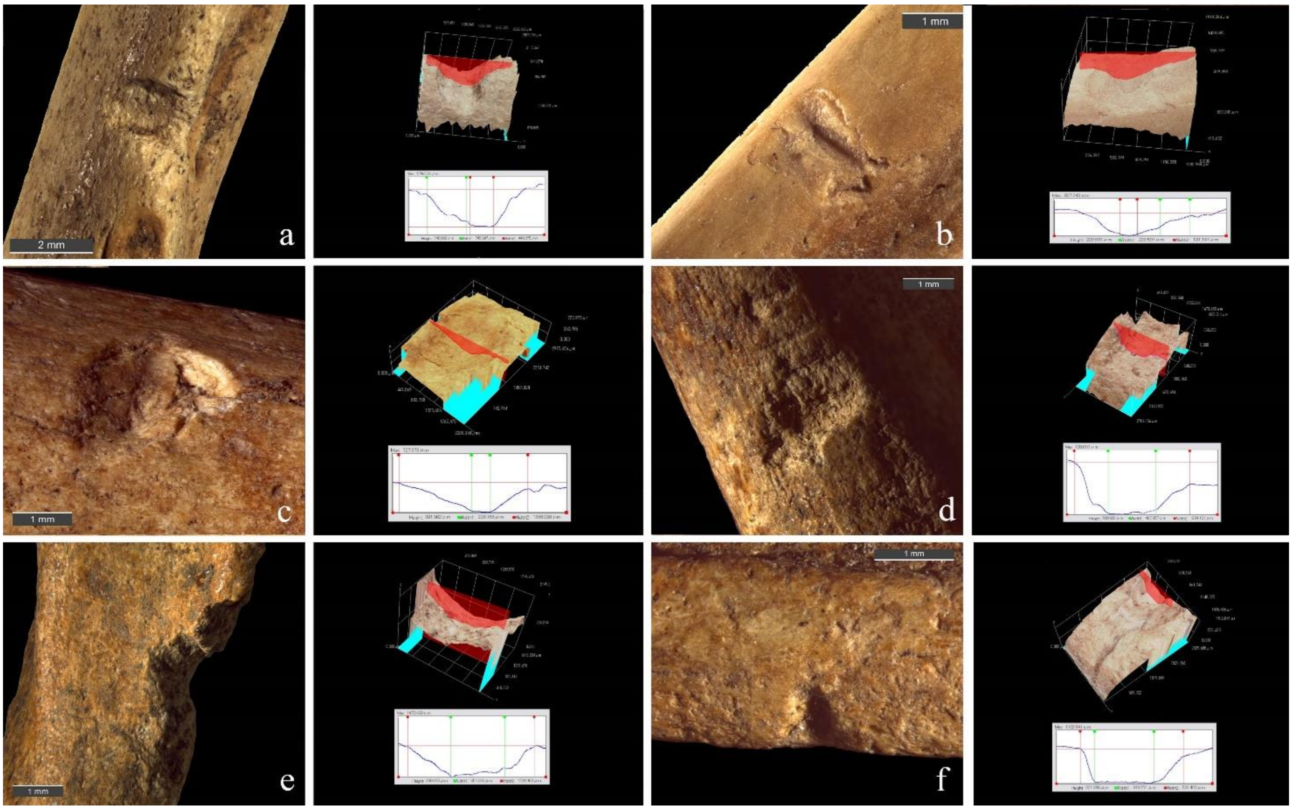

Supplementary Table S16: Results of Mann-Whitney U-test between the analysed samples; dm: archaeological drags on marmots; dc: experimental drags on coypus; cm: archaeological cut marks on marmots; cc: experimental cut marks on coypus. DC: depth of cut; BF: breadth at the floor of the cut; BT: breadth at the top of the cut; OA: opening angle; RTF: ratio between the breadth at the top and the breadth at the floor; RTD: ratio between breadth at the top and depth.

	DC dm	DC dc	DC cm	DC cc
DC dm	0	0,5957	3,8E-08	3,8E-07
DC dc		0	6,0E-05	1,6E-04
DC cm			0,0	0,1
DC cc				0,0
	BF_dm	BF_dc	BF_cm	BF_cc
BF_dm	-	0,01696	3,8E-10	6,5E-10
BF_dc		-	1,3E-04	3,0E-04
BF_cm			-	0,1
BF_cc				-

	BT_dm	BT_dc	BT_cm	BT_cc
BT_dm	-	0,2224	1,4E-09	4,7E-09
BT_dc		-	1,3E-04	2,7E-04
BT_cm			-	0,1
BT_cc				-
	OA_dm	OA_dc	OA_cm	OA_cc
OA_dm	-	1	0,9	0,4
OA_dc		-	0,6	0,3
OA_cm			-	0,1
OA_cc				-
	RTF_dm	RTF_dc	RTF_cm	RTF_cc
RTF_dm	-	0,0796	3,4E-08	1,2E-07
RTF_dc		-	4,5E-02	4,3E-02
RTF_cm			-	0,6
RTF_cc				-
	RTD_dm	RTD_dc	RTD_cm	RTD_cc
RTD_dm	-	0,4259	0,2	0,1
RTD_dc		-	0,8	0,5
RTD_cm			-	0,4
RTD_cc				-

Supplementary Table S17: Raw data of experimental drags on coypus, experimental cut marks on coypus, archaeological drags on marmots and archaeological cut marks on marmots. DC: depth of cut; BF: breadth at the floor of the cut; BT: breadth at the top of the cut; GD: greatest distance from the middle of the floor to the edges; SD: smallest distance from the middle of the floor to the edges; OA: opening angle.

Group	DC	BF	BT	GD	SD	OA	Name	Bone element
Archaeological Drags - Marmot	391,6	229	1577	1003,1	573,9	122,4	PR_3282_femore	Femur
Archaeological Drags - Marmot	492,3	803,7	1383,4	884,7	498,7	71,9	PR_2949_Femore	Femur
Archaeological Drags - Marmot	189,6	292,4	434,9	232,3	202,6	85,8	PR_2872_ulna	Ulna
Archaeological Drags - Marmot	321,4	319,2	554,4	331,7	222,7	40,8	PR_2621_1_ulna	Ulna
Archaeological Drags - Marmot	467	687,2	984,3	508,2	476,1	39,4	PR_652_femore	Femur
Archaeological Drags - Marmot	1333,4	792,8	1206,9	640,2	566,7	19,7	PR_4351_Tacca_coxale	Innominate
Archaeological Drags - Marmot	350,6	654	1529	846,2	682,8	91,9	PR_4538_coxale	Innominate
Archaeological Drags - Marmot	374,1	739,3	1080	569,5	510,5	54,8	PR_1624_omero	Humerus
Archaeological Drags - Marmot	78,8	101,7	352,5	179,4	173,1	120,3	PR_2020_radio	Radius
Archaeological Drags - Marmot	124,5	360,9	894,9	511,5	383,4	142,5	PR_2412_ulna	Ulna
Archaeological Drags - Marmot	257,5	359	898	482	416	75,4	PR_629_femore	Femur
Archaeological Drags - Marmot	308,1	622,5	1398,5	743,6	654,9	115,7	PR_1584_omero	Humerus
Archaeological Drags - Marmot	320,2	508,8	999,2	577,3	421,9	83,3	PR_8317_ulna	Ulna

Archaeological Drags - Marmot	610	204,5	2014	1256	758	106,6	PR8316Radio	Radius
Archaeological Drags - Marmot	147,8	233,7	696,4	386,3	310,1	122	PR8318Radio	Radius
Archaeological Drags - Marmot	229	131	855	468	387	109	PR1756Radio	Radius
Archaeological Drags - Marmot	1538	2412	2701	1352	1349	10	PR2243Radio	Radius
Archaeological Drags - Marmot	746	440,3	1843	1035	808	65,6	PR8315Radio	Radius
Archaeological Drags - Marmot	1131	753	2191	1240	951	39,9	PR8297Mandibola	Mandible
Archaeological Drags - Marmot	179	240,5	951	477	474	133,6	PR8303Radio	Radius
Archaeological Drags - Marmot	175,5	278,8	658,7	392,3	266,4	81,9	PR8312Ulna	Ulna
Archaeological Cut marks - Marmot	50,5	10,4	143,9	78	65,9	90,9	PR160Ulna_a	Radius
Archaeological Cut marks - Marmot	48,3	20,4	226,1	174,3	51,8	98,2	PR160Ulna_b	Radius
Archaeological Cut marks - Marmot	72,7	15,7	208,3	112	96,3	84,8	PR2275Radio_a	Radius
Archaeological Cut marks - Marmot	12	14,6	71,4	41,4	30	129,8	PR2275Radio_b	Radius
Archaeological Cut marks - Marmot	10,3	6,9	62,8	43,9	18,9	120,1	PR2275Radio_e	Radius
Archaeological Cut marks - Marmot	76	23,6	233,4	128,2	105,2	114,4	PR227Scapola_a	Scapula
Archaeological Cut marks - Marmot	50,7	12,5	174,3	88,3	86	94,9	PR227Scapola_b	Scapula
Archaeological Cut marks - Marmot	230,7	20,9	459,4	360,3	99,1	64,9	PR25Scapola_a	Scapula
Archaeological Cut marks - Marmot	72,9	24,4	206	102,8	103,2	83,2	PR412Clavicola_a	Clavicle
Archaeological Cut marks - Marmot	241,5	20,9	511,6	310,8	200,8	82,2	PR412Clavicola_b	Clavicle
Archaeological Cut marks - Marmot	94,9	16,4	216	127,4	88,6	73	PR413Clavicola_a	Clavicle
Archaeological Cut marks - Marmot	176	99,5	592	315,9	276,1	88,1	PR4544Ischio_a	Innominate
Archaeological Cut marks - Marmot	134	28,3	426,4	247,3	179,1	88,7	PR4544Ischio_b	Innominate
Archaeological Cut marks - Marmot	72,5	8,7	113,7	79,3	34,4	54,8	PR4545Pube_a	Innominate
Archaeological Cut marks - Marmot	34,6	28,3	130,9	75,8	55,1	96,6	PR4545Pube_b	Innominate
Archaeological Cut marks - Marmot	189,3	19	423	223	200	52,6	PR4545Pube_b	Innominate
Archaeological Cut marks - Marmot	126,2	51,4	167,7	100,2	67,5	43,8	PR4667Omero_a	Humerus
Archaeological Cut marks - Marmot	65,8	3,4	263,7	194,2	69,5	83,7	PR4667Omero_b	Humerus
Archaeological Cut marks - Marmot	34,4	20,9	119	68,3	50,7	106	PR4667Omero_c	Humerus
Archaeological Cut marks - Marmot	162,6	49,8	359,6	214,2	145,4	55,8	PR4667Omero_d	Humerus
Archaeological Cut marks - Marmot	176,3	117,8	361,4	212,9	148,5	61,4	PR4667Omero_e	Humerus
Archaeological Cut marks - Marmot	63,4	82,9	255,5	158,7	96,8	97,4	PR4667Omero_f	Humerus
Archaeological Cut marks - Marmot	96,1	11,4	152,5	80	72,5	54,5	PR581Omero_a	Humerus
Archaeological Cut marks - Marmot	46,6	15	91,5	52,5	39	71,8	PR581Omero_b	Humerus
Archaeological Cut marks - Marmot	59,5	16,6	136,3	81,4	54,9	74,4	PR6074Costa_a	Rib
Archaeological Cut marks - Marmot	159	16,1	548,4	379,8	168,6	113,7	PR6074Costa_b	Rib
Archaeological Cut marks - Marmot	79,9	18,5	216,7	112	104,7	86,2	PR6346Mandibola_a	Mandible
Archaeological Cut marks - Marmot	60,3	20,45	322,8	166,8	156	104,1	PR6346Mandibola_b	Mandible
Archaeological Cut marks - Marmot	60	20	156,5	80	76,5	85,9	PR6349Mandibola_a	Mandible
Archaeological Cut marks - Marmot	77,6	64,3	231,7	127,3	104,4	81,9	PR6349Mandibola_b	Mandible
Archaeological Cut marks - Marmot	79,4	12,1	225,8	122,6	103,2	92,8	PR6350Mandibola_c	Mandible
Archaeological Cut marks - Marmot	79	20,3	142,7	94	48,7	62,6	PR6350Mandibola_d	Mandible
Archaeological Cut marks - Marmot	69,2	17,2	181	95,8	85,2	86,7	PR6350Mandibola_e	Mandible
Archaeological Cut marks - Marmot	126,5	20,1	229,7	134,9	94,8	69,9	PR6350Mandibola_f	Mandible
Archaeological Cut marks - Marmot	147,6	25,3	415,6	279,1	136,5	86,2	PR78Tibia_a	Tibia
Archaeological Cut marks - Marmot	129,9	23,3	272,7	189,3	83,4	71	PR78Tibia_b	Tibia
Archaeological Cut marks - Marmot	71,6	47,4	231,8	143,1	88,7	82,8	PR8111Premascellare_a	Skull
Experimental Drags - Coypu	442,9	548	1448	791	657	56,2	OST00001_001 Nutria1Giorno1Omerodx	Humerus
Experimental Drags - Coypu	455	62	1366	712	654	116,5	OST00001_002 Nutria1Giorno1Radiodx	Radius

Experimental Drags - Coypu	268,7	235	1034	642	392	84,8	OST00006_003 Nutria1Giorno3Radio	Radius
Experimental Drags - Coypu	209	47,6	660,8	334	326,8	100	OST00005_001 Nutria3Giorno2Coxale	Innominate
Experimental Drags - Coypu	260,9	243,3	668,8	364,6	304,2	101	OST00006_001 Nutria 1 Incisivo	Incisor
Experimental Drags - Coypu	243	194,8	648,2	392,4	255,8	93,8	OST00006_001 Nutria_1_Incisivo	Incisor
Experimental Drags - Coypu	214,8	182,7	303,6	160,2	143,4	35,5	OST00007_001 Nutria 2 T72 coxale	Innominate
Experimental Cut marks - Coypu	403	34	601	409	192	51,9	OST00009_012 Nutria1Caudale	Caudal vertebra
Experimental Cut marks - Coypu	161,3	19	466,5	401	65,5	80,3	OST00009_002_a Nutria1Clavicola_a	Clavicle
Experimental Cut marks - Coypu	24	13,7	84	56,3	27,7	91	OST00009_002_b Nutria1Clavicola_b	Clavicle
Experimental Cut marks - Coypu	48,9	22,4	122,7	92,8	29,9	73	OST00009_002_c Nutria1Clavicola_c	Clavicle
Experimental Cut marks - Coypu	136	21,9	290	150	140	66,6	OST00009_007_a Nutria1CoxaleDx_a	Innominate
Experimental Cut marks - Coypu	119,3	16,8	209,4	121	88,4	70,9	OST00009_007_b Nutria1CoxaleDx_b	Innominate
Experimental Cut marks - Coypu	63,9	94,7	255,6	140,5	115,1	97,6	OST00009_009_a Nutria1FibulaA_a	Fibula
Experimental Cut marks - Coypu	127	58,8	398,5	253,5	145	80,7	OST00009_009_c Nutria1FibulaA_b	Fibula
Experimental Cut marks - Coypu	95,3	41,9	196,6	135,5	61,1	64,4	OST00009_009_c Nutria1FibulaA_c	Fibula
Experimental Cut marks - Coypu	113	61,2	274	162	112	68,9	OST00009_010_a Nutria1FibulaB_a	Fibula
Experimental Cut marks - Coypu	64,4	14,7	197,7	109,5	88,2	90	OST00009_010_b Nutria1FibulaB_b	Fibula
Experimental Cut marks - Coypu	45,5	6,3	153,2	92,2	61	118	OST00009_010_c Nutria1FibulaB_c	Fibula
Experimental Cut marks - Coypu	133	26,6	437	302	135	100	OST00009_001 Nutria1Mandibola	Mandible
Experimental Cut marks - Coypu	111,5	47	391	302	89	94,7	OST00009_003 Nutria1OmeroA	Humerus
Experimental Cut marks - Coypu	199	27,9	469	370	99	53,7	OST00009_004 Nutria1OmeroB	Humerus
Experimental Cut marks - Coypu	91,5	145,9	485,1	334,1	151	101,2	OST00009_005_a Nutria1Radiodx_a	Radius
Experimental Cut marks - Coypu	84,5	21,1	142,75	57,6	85,15	59,9	OST00009_005_b Nutria1Radiodx_b	Radius
Experimental Cut marks - Coypu	44	14,3	106,7	68,8	37,9	94,3	OST00009_005_c Nutria1Radiodx_c	Radius
Experimental Cut marks - Coypu	142,8	16	296,1	150,8	145,3	87,7	OST00009_005_d Nutria1Radiodx_d	Radius
Experimental Cut marks - Coypu	109,5	35,6	235	150	85	60,6	OST00009_006 Nutria1Radiosin	Radius
Experimental Cut marks - Coypu	161,1	83,2	393,9	228,1	165,8	86,6	OST00009_008_a Nutria1Tibiasin_a	Tibia
Experimental Cut marks - Coypu	113,7	77,3	410,5	270,7	139,8	100,7	OST00009_008_b Nutria1Tibiasin_b	Tibia
Experimental Cut marks - Coypu	117,5	21,2	308	216,6	91,4	62,4	OST00010_002_a Nutria2Costa2_a	Rib
Experimental Cut marks - Coypu	138,8	15,5	271	177,7	93,3	44,1	OST00010_002_b Nutria2Costa2_b	Rib
Experimental Cut marks - Coypu	160,1	29,8	293	134,3	158,7	47,6	OST00010_003_a Nutria2Costa3_a	Rib
Experimental Cut marks - Coypu	106,3	19,9	179,1	125,2	53,9	79,5	OST00010_003_b Nutria2CostA3_b	Rib
Experimental Cut marks - Coypu	76,8	52	359,9	274,6	85,3	83,8	OST00010_004 Nutria2CostolaDorsale	Rib
Experimental Cut marks - Coypu	110	62,5	606,5	347,1	259,4	115,4	OST00010_005_a Nutria2FemoreCaputDx_a	Femur
Experimental Cut marks - Coypu	137,6	21,8	193,4	124,8	68,6	61	OST00010_005_b Nutria2FemoreCaputDx_b	Femur
Experimental Cut marks - Coypu	95,9	37,2	194,2	125,8	68,4	56,8	OST00010_005_c Nutria2FemoreTrocantereDx_a	Femur
Experimental Cut marks - Coypu	325	28,7	464,5	326,5	138	60	OST00010_005_d Nutria2FemoreTrocantereDx_b	Femur
Experimental Cut marks - Coypu	96,97	76,14	328,9	227,3	101,6	81,1	OST00010_005_e Nutria2FemoreTrocantereDx_c	Femur
Experimental Cut marks - Coypu	41,8	10,5	183,4	161,5	21,9	62,4	OST00010_001_a Nutria2ParietaleSin_a	Skull
Experimental Cut marks - Coypu	32,9	12,8	70,5	53,9	16,6	74,7	OST00010_001_b Nutria2ParietaleSin_b	Skull
Experimental Cut marks - Coypu	144,9	15,8	277,1	213,5	63,6	55,7	OST00009_011 Nutria1Sacro1	Sacrum

Supplementary Figure S18: Location of the archaeological PIMs recognised on marmot bones from Pradis Cave. The drawing of the *Marmota marmota* skeleton was made by Cédric Beauval and Carine Tomé Carpentier, and taken from ArcheoZoo.org (available at: https://www.archeozoo.org/archeozootheque/picture/2892-marmota_marmota/category/144-rongeurs_langen_rodents_lang_langes_roedores_lang_ under the following licence https://creativecommons.org/licenses/by-sa/4.0/) and then modified by us with the addition of the location of the archaeological PIMs.

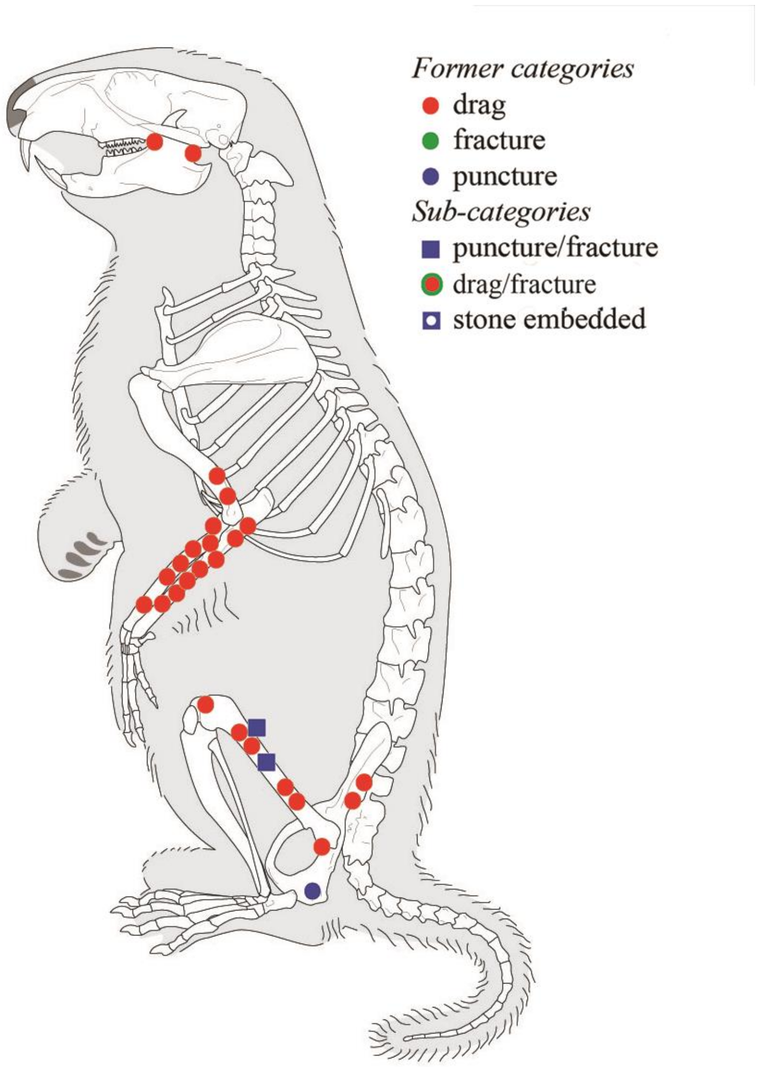

© 2005 ArcheoZoo.org / Cédric Beauval, Carine Tomé Carpentier
After : Morel, Ph. & Muller, W. Hauterive-Champréveyres 11 : Un campement magdalénien au bord du lac de Neuchâtel : étude archéozoologique (secteur 1). *Archéologie Neuchâteloise* **23**, 74-82 (1997)

Supplementary Figure S19: Experimental framework.

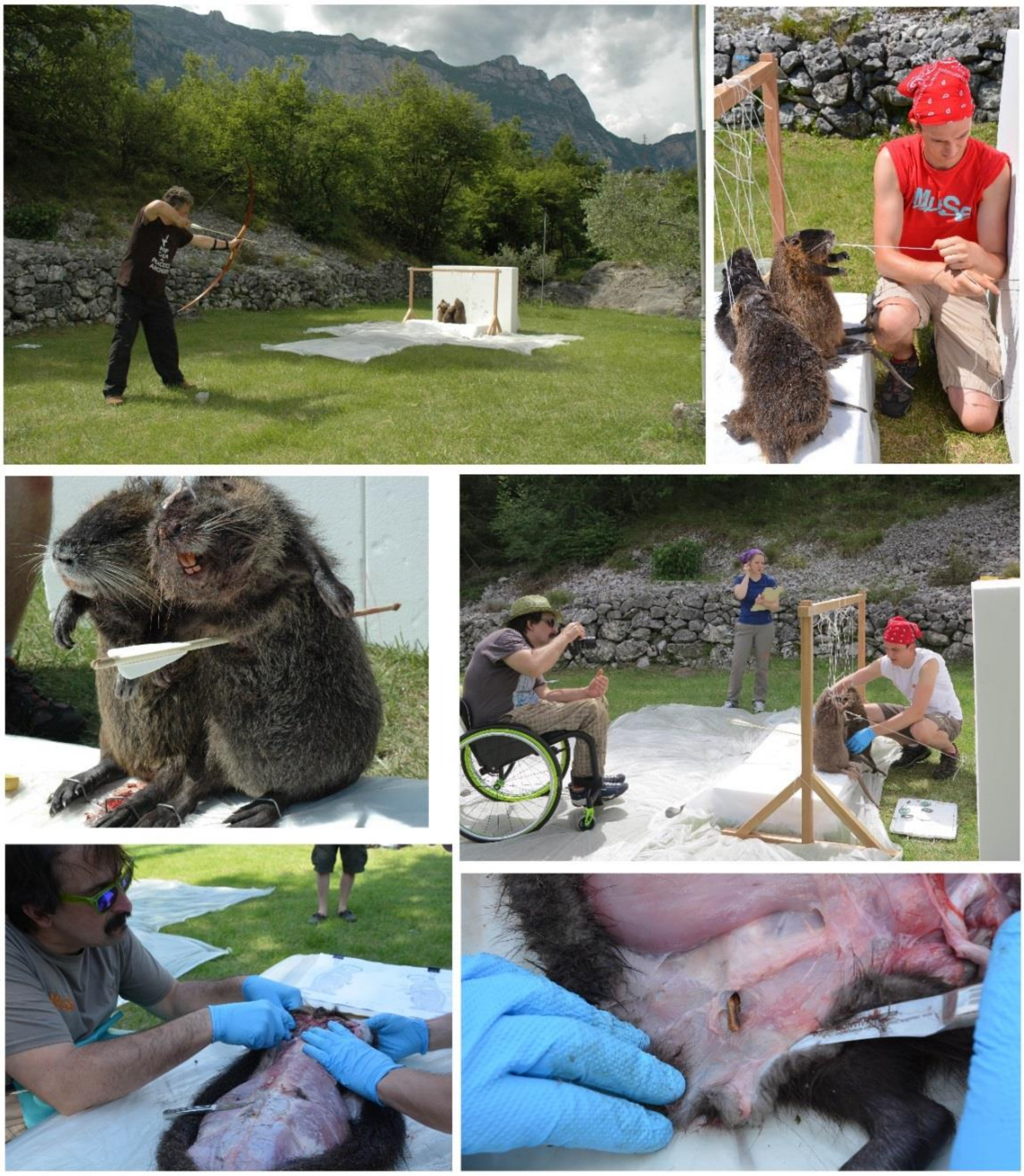

Supplement: Supplementary file 1 — Supplementary Information. [file 41598_2020_66044_MOESM1_ESM.pdf]
